# Using Dendritic Heat Maps to Simultaneously Display Genotype Divergence with Phenotype Divergence

**DOI:** 10.1371/journal.pone.0161292

**Published:** 2016-08-18

**Authors:** Matthew Kellom, Jason Raymond

**Affiliations:** School of Earth and Space Exploration, Arizona State University, Tempe, Arizona, United States of America; Massey University, NEW ZEALAND

## Abstract

The advancement of techniques to visualize and analyze large-scale sequencing datasets is an area of active research and is rooted in traditional techniques such as heat maps and dendrograms. We introduce dendritic heat maps that display heat map results over aligned DNA sequence clusters for a range of clustering cutoffs. Dendritic heat maps aid in visualizing the effects of group differences on clustering hierarchy and relative abundance of sampled sequences. Here, we artificially generate two separate datasets with simplified mutation and population growth procedures with GC content group separation to use as example phenotypes. In this work, we use the term phenotype to represent any feature by which groups can be separated. These sequences were clustered in a fractional identity range of 0.75 to 1.0 using agglomerative minimum-, maximum-, and average-linkage algorithms, as well as a divisive centroid-based algorithm. We demonstrate that dendritic heat maps give freedom to scrutinize specific clustering levels across a range of cutoffs, track changes in phenotype inequity across multiple levels of sequence clustering specificity, and easily visualize how deeply rooted changes in phenotype inequity are in a dataset. As genotypes diverge in sample populations, clusters are shown to break apart into smaller clusters at higher identity cutoff levels, similar to a dendrogram. Phenotype divergence, which is shown as a heat map of relative abundance bin response, may or may not follow genotype divergences. This joined view highlights the relationship between genotype and phenotype divergence for treatment groups. We discuss the minimum-, maximum-, average-, and centroid-linkage algorithm approaches to building dendritic heat maps and make a case for the divisive “top-down” centroid-based clustering methodology as being the best option visualize the effects of changing factors on clustering hierarchy and relative abundance.

## 1.0 Introduction

Advances in sequencing technology and–omics research has led to rapid growth in sequencing datasets, and techniques to visualize and analyze the data are struggling to keep up. New avenues of research that expand on traditional techniques are being explored with much room for further advancement, as software development attempts to meet the demands of elucidating important aspects of such large and complex datasets like metagenomes or metatranscriptomes. One of the most fundamental steps for analysis of any sequence dataset is annotation and classification into hierarchies, which can be achieved via sequence comparison tools such as USEARCH, the Ribosomal Database Project (RDP) Classifier, the Basic Local Alignment Search Tool (BLAST), RapSearch2, and Phylosift [1,2,3,4,5]. While all of these tools do well at the annotation of sequences, there is a well-known classification bias that comes with limited databases that do not contain true representatives of every sequence [6]. The development of more efficient and accurate comparison tools is an area of active research, and understanding the results of these tools in the context of *in vivo* dynamics is of great interest. MEGAN is one of the more well-known software options for metagenomic analysis and visualization of sequence comparison results, as are some web-based platforms such as MG-RAST, METAREP, and Krona [7,8,9,10]. The fundamentals of traditional techniques such as heat maps and dendrograms are at the root of all these recent software advances, where hierarchy and relative abundance are represented through branching and value indicators, respectively. Here, we explore a method of creating dendritic heat maps (DHMs) that combines heat maps and dendrograms in order to visualize phenotype divergence alongside genotype divergence. We use the term phenotype to represent any feature by which groups can be separated (e.g. physical traits, locations, growth conditions, etc.). Importantly, DHMs are not limited to sequence data and can be used to describe changes in group inequity and clustering hierarchy for any data that can be hierarchically ordered and compared (e.g. microarrays, phylotype counts, species richness, etc). However, here we exclusively examine their application toward sequence datasets.

Heat maps are useful for comparing data across a range of possible states, allowing viewers to intuitively see differences and similarities in data subset responses. Canonically, each data point is expressed as a color, with hue intensity representing its bin response, or position within the data range [11]. Likewise, sections on a heat map can also represent bins of data, where individual data points have been grouped together based on similarity and their corresponding heat map color is a result of their combined response [12]. Clustering, especially of DNA, RNA, or protein sequences, is commonly used for data binning and is based on sequence identity or homology. For instance, 16S rRNA heat maps are frequently used to compare relative abundances of sequences between multiple samples, allowing visualization of the presence and absence of taxa across samples or populations [13,14,15]. With genomic and transcriptomic data, binning and visualization on heat maps can be used to compare and contrast gene and transcript abundances, with the most common use of heat maps being visualization of changes in gene expression across different sample treatments or conditions [16,17,18].

However, heat map bin response for a data point can change depending on the level of specificity, defined by the cutoff level at which that data is binned and visualized. By altering the binning specificity level, data bin assignment can be rearranged, potentially changing their heat map bin response. Traditional heat maps only work at a single specificity level and limit viewers to one representation of the data. For instance, heat maps that depict clustering of 16S rRNA gene sequences are typically done at a 97% identity level. Choosing an appropriate specificity level then becomes crucial to not obscuring the important features of the data with bins that are too specific or too broad. For example, bins that are too specific for a transcriptome dataset can result in fractured count information with many clusters identified as the same target sequence. Conversely, bins that are too broad for a transcriptome dataset can result in clusters containing multiple transcript groups. Both of these scenarios can be problematic if only one cutoff level is being displayed.

Our motivation for this work is to visualize sequence dynamics in a way that captures important variations in the data and is scalable across a large range of data sizes. We also wanted to explore a technique that is independent of annotation and instead performs analysis and visualization of sequence information, leaving annotation as a final step, since sequence reference databases are dependent on the quality and focus of previous work. To these ends we improve on current techniques in three areas:

1. Freedom to scrutinize specific clustering levels across a range of cutoffs.
2. Ability to track changes in state across multiple levels of sequence clustering specificity.
3. Ease to visualize how deeply rooted changes in state are in a data set.

DHMs are particularly useful where similarities in a dataset occur across a multitude of scales, such as in homology-based clustering of the large number of sequences found within a microbial community. Because the sequences within a complex community can range from being extremely well conserved to very poorly conserved, our method will allow the simultaneous visualization of homology clusters at many different cutoffs. Importantly, tracking changes in relative abundance bin response can be particularly useful for observing the levels at which genotypic divergence (cluster branching) correlates with phenotypic divergence (differing heat map bin response) for a population.

To demonstrate this approach, we generate artificial datasets that use simplified mutation and growth processes in biological communities. The first dataset starts with 100 identical 100-bp DNA fragments which all mutate with random single base substitutions over fifteen iterations. The second dataset is used exclusively with the “top-down” method due to its size and begins with a 100-bp DNA fragment, allowing it to mutate and duplicate, then iterating the mutate-and-duplicate process on progeny DNA. The end result is a population of tens-of-thousands of DNA molecules derived from a common ancestor, each showing varying degrees of conservation. Clustering and visualization of changes of state are used to track relative abundance bin responses for populations of different nucleotide usage (GC content) as various subpopulations evolve for both datasets. Using these simulated datasets, we discuss the potential of DHMs to describe data across varying levels of complexity.

## 2.0 Methods

### 2.1 Sequence Generation

#### Mutation Lineage Data Set

Random base substitutions were performed on a set of 100 artificially-generated 100-bp DNA sequences over fifteen iterations. Base substitutions are allowed to occur at the same position more than once. At iteration zero, all 100 sequences are identical with 50% GC content; at iteration fifteen they have the least homology with 15 random base substitutions having occurred in each sequence. At each iteration all 100 sequences were grouped based on ≤ or >50% GC content and output to FASTA files. The ≤ or >50% GC content group sizes were kept relatively even by restarting the sequence generation if the count difference between the two groups exceeded 20 sequences (|Group1-Group2|>20) (20% of the total dataset). This group evening was done to ensure a good representation of each group for an effective demonstration of DHMs.

#### Population Growth Data Set

Additional FASTA files of fifteen generations started from a single randomly-generated sequence, which was subsequently propagated by duplicating each sequence once with a random base substitution and once without at each generation of population growth, reaching 2^15^ sequences after fifteen generations. The artificial growth process created fifteen separate, but related, datasets to demonstrate the ability of top-down centroid-based clustering to handle larger datasets for DHM construction. The artificially-generated 100-bp DNA sequences were grouped based on ≤ or >50% GC content. The ≤ or >50% GC content group sizes were kept relatively even by restarting the sequence generation if the difference between the number of sequences for the two groups became greater than 5% of the total amount of sequences (|Group1-Group2|>Total*0.05). This group evening was done to ensure a good representation of each group for an effective demonstration of DHMs.

### 2.2 Clustering

#### Bottom-up agglomerative clustering

For each incremental 0.01 fractional identity (“-id”) cutoff between 0.75 and 1.0, clustering of sequences was performed using USEARCH (version 8.0.1517_i86linux64) (“-cluster_agg”) with linkages min, max, and avg.

#### Top-down divisive clustering

**Fig 1:**
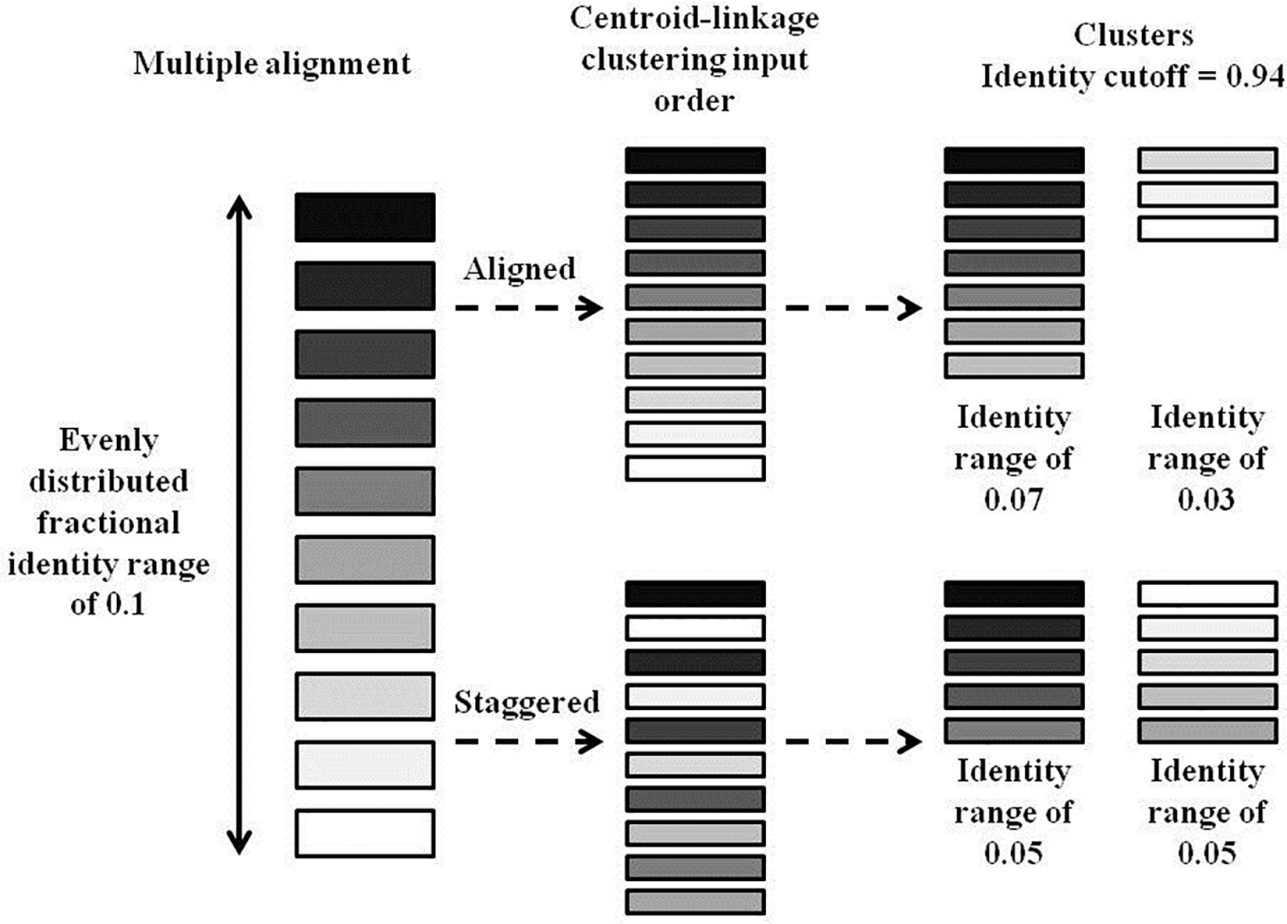
Sequence input ordering. Graphical representation of the binning effect of using alignment-ordered versus staggered sequence input order for “top-down” centroid-based clustering. Shaded rectangles represent sequences, where the shade consistently portrays a specific sequence throughout the diagram. The multiple alignment on the left shows each of the sequences ordered based on fractional identity, where nearby sequences are more closely related than distant ones, and distributed evenly across a fractional identity range of 0.1. For both aligned and staggered input ordering, sequences are read from top to bottom by the UCLUST algorithm of USEARCH and either placed in a cluster that has the best match to the centroid sequence above the given identity cutoff, or is made the centroid sequence of a new cluster if a match cannot be found. In this diagram, centroid sequences are the top sequences of each cluster. With the aligned input order, it is shown that some sequences can be binned in clusters that do not contain their closest centroid match. The staggered input places sequences in correct bins essentially by first defining all centroid sequences.

For the each 0.01 -id cutoff between 0.75 and 1.0, clustering of sequences was performed using the USEARCH UCLUST algorithm (version 7.0.1090_i86linux64) (“-cluster_fast”), which performs centroid-based clustering. Counts of duplicate sequences were recorded with “-derep_fulllength” [1]. Clustering at each cutoff -id was done in a stepwise fashion, starting from the initial FASTA file for 0.75, then using the clusters from the previous cutoff as inputs for 0.76–1.0. Since the USEARCH command “-cluster_fast” performs centroid-based clustering in the order of the input FASTA file, input sequences were first multiple aligned using Clustal Omega (described later) and arranged to ensure that the most distantly related and potentially cluster splitting “centroid” sequences were listed first in a staggered order (conceptualized in Fig 1). This ordering process was performed by a script that reads from opposite ends of the multiple alignment and is unnecessary for the “bottom-up” approaches since binning is done through the use of a distance matrix. A brief example of the top-down clustering is available as S1 File.

### 2.3 Alignment

Clustal Omega (Version 1.2.0) with tree-output ordering and default alignment parameters and heuristics was used for multiple alignment of FASTA files [19]. Manipulation of the multiple alignment and clustering files were performed via Perl scripts, which are available in S2 and S3 Files. This multiple alignment was used to order the lowest -id cutoff (0.75) clusters, then the sequences contained within them. For each -id cutoff from 0.76 to 1.0, the order was determined by the arrangement of the previous -id cutoff. In the “top-down” method, this alignment was also used to order the clustering input files in a staggered fashion (Fig 1). Alignment is important for preserving the radial position of each sequence at each cutoff level/ring in the DHMs so that the position of any given sequence is preserved from its center out to its circumference. Starting the clustering cutoff range at a minimum value of 0.75 was done because the USEARCH manual states that the UCLUST algorithm is effective at identities of ~75% and above for nucleotide sequences, but dendritic heat maps in general are not limited to this cutoff range and should aim to show as large a clustering cutoff range as possible.

### 2.4 Dendritic Heat Map Construction

Visualization of the clusters at each cutoff and their arrangements was performed using the Perl package Circos (version 0.64) [20]. For each -id cutoff ring, cluster sizes are determined by number of sequences within each cluster. The heat map hues are partitioned to have gradual changes with twenty-three possible categories (two sequential 11-color Brewer palettes and white) and is determined by the logarithmic value of the ratio of sequences from each group, log((Group1 +1)/(Group2 +1)). The log of the ratio is used because in typical clustering datasets, the vast majority of the clusters are relatively small and a few are very large. Without using the log of the ratio, smaller clusters with an interesting bin response would be assigned a neutral hue and only the largest clusters would have the most luminous hues. A log transformation of the ratios puts all bin responses on a more relatable scale while still showing their distinctions in ratio. Red hue indicates relative abundance bin response toward Group 1(GC≤50%) and blue hue indicates relative abundance bin response toward Group 2 (GC>50%), with white indicating a neutral bin response. A red-white-blue color scheme was chosen here because it is more color-blind friendly, however any color scheme can be used. Hue luminosity corresponds to the strength of the heat map response. A darkly colored wedge at the 0° position acts as a key, displaying the opposite ends of the heat map hues possible for each ring. The minimum and maximum heat map values in this wedge are equal at their absolute values and are important for normalizing the hue distribution throughout the entire DHM. A brief example of DHM construction is available in S1 File.

## 3.0 Results/Discussion

### 3.1 Dendritic Heat Map

To make relationships that may emerge across hierarchical cutoffs more apparent, heat maps at each cutoff level are aligned, so that clusters may be directly compared across multiple cutoff levels. By aligning and clustering DNA molecules across these multiple cutoff levels, the aligned heat maps take on a radial dendrogram configuration. This is particularly useful, as the branching of a cluster into progressively more fine-grained clusters can be tracked and further annotated with heat map bin responses to reveal salient features of the clusters. To visualize DHMs, we make use of a Perl-based software package called Circos that was developed to address challenges in visualizing large genomic data sets and creates circular heat maps from position and value data [20]. In the case of DHMs, circular heat maps allow for the placement of exterior ring heat map bins to be fanned out, giving them more space than interior ring bins where the need for space is less critical. While a useful tool for generating images, Circos is illustration software that is dependent on user-formatted input files and is not designed to analyze raw data or arrange heat maps. Therefore, we have developed Perl scripts (available in S2 and S3 Files) to align and convert clustering data derived from large scale sequence datasets to Circos-ready input files.

**Fig 2:**
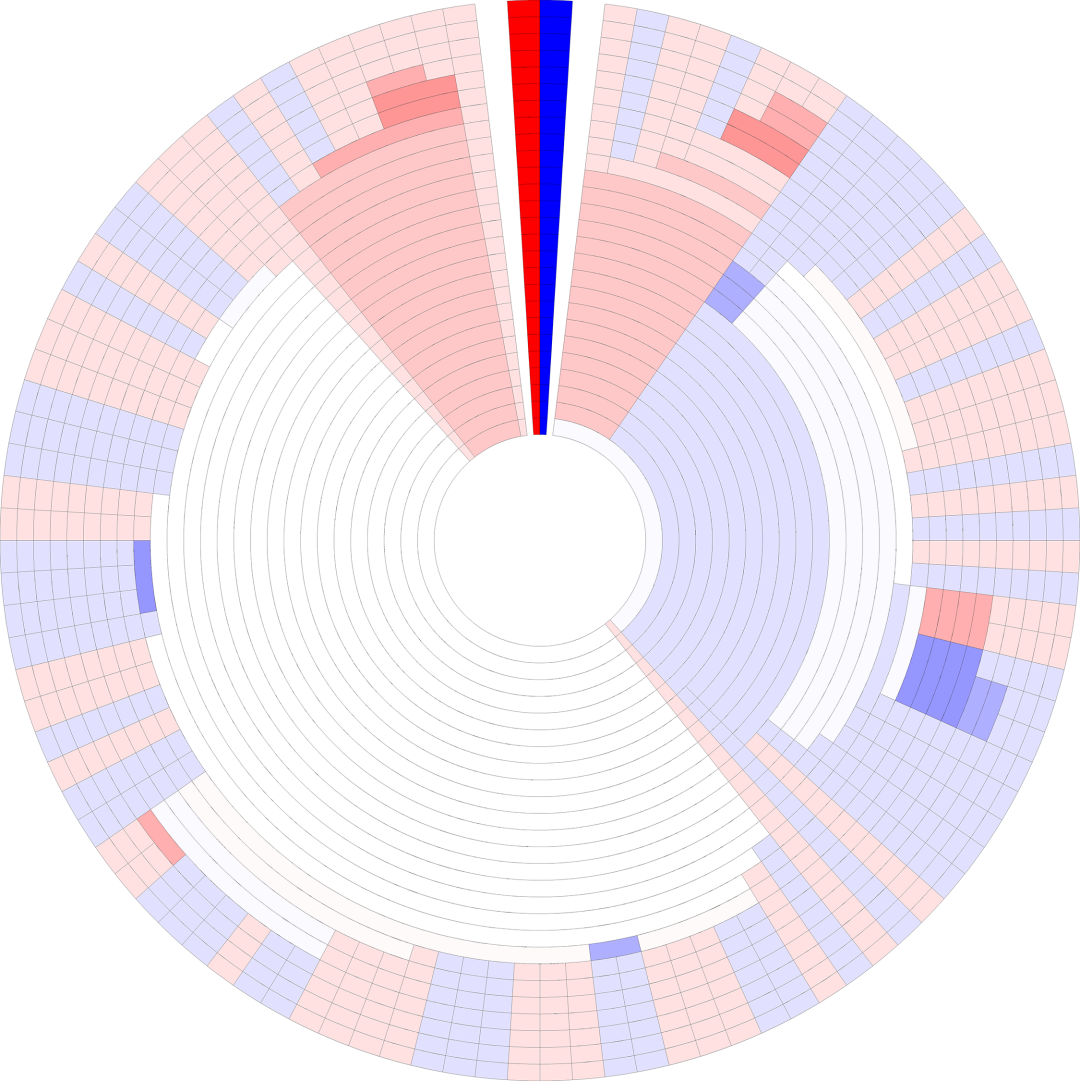
Dendritic heat map. Dendritic heat map representing the fifth mutation step of the simulated mutation lineage data generated as described in the methods and clustered using the average-linkage algorithm of the “bottom-up” method. The darkly colored wedge at the 0° position represents the minimum (red) and maximum (blue) possible heat map relative abundance bin responses, GC≤50% and GC>50% respectively. White space in the heat maps represents clusters with neutral bin response. Rings, starting at the center, represent clusters of sequences for identity cutoffs of 0.75 to 1.0. Clusters, including single-sequence clusters, are plotted in a radial range that is conserved from the clusters from which they were derived. High resolution versions of all DHMs in this manuscript are available in S2 and S3 Files.

Fig 2 shows the average-linkage DHM for the fifth mutation from the mutation lineage data set generated as described in the methods section. The feature that is immediately recognized is the heat map color variation in different regions of the figure, displaying the relative abundance bin response of GC content groups (neutral–white; Group 1 bin response (GC≤50%)–red; Group 2 bin response (GC>50%)–blue) for each cluster. The decision to use red and blue hues was aided with the use of ColorBrewer palettes (http://colorbrewer2.org/) to represent values as a colorblind-friendly alternative to the red-green color scheme that is a popular heat map motif [21]. However, RGB color codes were eventually used to select hues outside of ColorBrewer palettes. Navigating through the figure, the innermost ring represents clusters at the most lenient fractional identity cutoff of 0.75, stepping out in increments of 0.01 at each ring to a final identity cutoff of 1.0. Simultaneously displaying a range of heat maps that change with specificity level gives a more accurate view of how the data is skewed than any single heat map can provide individually, due to the fact that heat map bin response for a data point can change direction depending on the level of specificity at which the data is binned. In a natural data example, central rings with lower cutoffs would be associated with broad groupings such as phyla or gene superfamilies, while the increasingly strict cutoffs at the peripheral rings would represent more specific identifiers (e.g. same genus/species, gene families/subfamilies). Studies that involve natural data sets might not be grouped based on GC content, but rather some separation that is relevant to the questions being asked or conditions being measured (and that could be easily substituted into a DHM). For our purposes, using GC content as a phenotype is a convenient method for creating and tracking relative abundance bin responses of two distinct groups while also forcing a correlation between genotype and phenotype for these artificially generated datasets.

The more subtle feature of Fig 2 is the dendrogram-like layout of the figure rings. The aligned configuration of the rings preserves the relationships of a dendrogram, where nearby clusters and sequences are closer relatives than distant clusters. The more central in the diagram a cluster divergence occurs, the more distantly related those clusters are to one another. Since each ring is aligned to adjacent rings, large clusters gradually divide into smaller and more specific clusters moving out from the center. In Fig 2, many of the clusters in the exterior rings contain single-sequence clusters that have a conserved radial range from the more interior clusters they derive from. Some clusters contain sequences that are highly conserved and their membership does not change over a wide range of clustering cutoffs, as seen at around the 11 o’clock position of Fig 2. Also important to point out are cases where bin response changes in direction and not just intensity. At about the 3 o’clock position of Fig 2, interior rings contain a large blue (mostly GC>50% sequences) cluster that gradually divides toward the exterior rings. These clusters divide, some of their bin responses change from blue (mostly GC>50% sequences), to the neutral white, to red (mostly GC≤50% sequences). The darkly colored wedge at the 0° position represents the minimum (red) and maximum (blue) possible heat map relative abundance bin responses, GC≤50% and GC>50% respectively. This wedge at the 0° position serves two functions. First, the wedge serves as a legend for the minimum and maximum heat map bin responses. Second, the wedge separates the most distantly related clusters at opposite ends of the DHM. Without a separation, it may be easy to confuse the clusters at opposite ends of the dendrogram layout as closely related. White space in the heat maps represents clusters with neutral bin response.

Overlaying phenotype shifts on genotype divergence creates a way to visually compare how deeply rooted observed phenotype ratios are across multiple genotypes. Displaying how deeply rooted a response is can be informative in many comparative studies that seek to better understand both the general and more finely detailed structures of the data by elucidating divergence points. Work that focuses on multi-scale genomic changes, such as experimental evolution of microbial or viral populations, would benefit from the visualizations of DHMs [22,23].

### 3.2 Bottom-up Hierarchical Clustering

For agglomerative, or “bottom-up,” approaches where clusters are joined by incrementally decreasing the sequence identity required to bin sequences together, we contrasted minimum-, maximum-, and average-linkage algorithms, all common graph metrics. Briefly, these methods differ in how connections between cluster elements (i.e. the edges connecting various nodes within a cluster) affect cluster membership. Minimum-linkage, sometimes referred to as nearest neighbor, only requires a single edge between two clusters above a specified cutoff before they can be joined, regardless of the other edge relationships [24,12]. Maximum-linkage, sometimes referred to as complete-linkage or farthest neighbor, requires all elements of a cluster to have a cutoff-agreeing edge to all elements of a joining cluster [25]. Average-linkage, sometimes referred to as mean linkage or unweighted pair group method with arithmetic mean (UPGMA), requires the mean of all cluster element edge distances to meet the clustering cutoff before they can be joined [26,25]. However, all of these agglomerative linkage methods yield relatively poor results when compared to a divisive method, mainly due to cluster joining requirements (discussed in detail below) as opposed to cluster splitting requirements.

**Fig 3:**
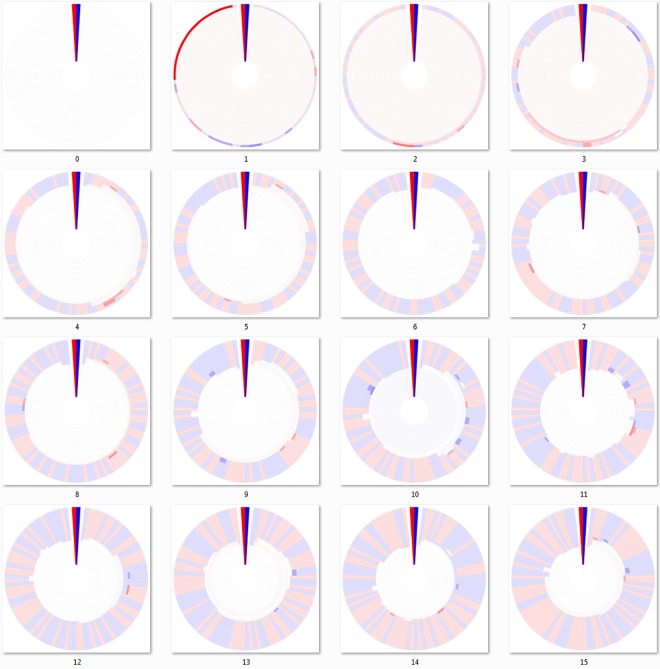
Dendritic heat maps from bottom-up minimum-linkage hierarchical clustering of a mutating population. Dendritic heat maps representing 0 through 15 mutations of the simulated mutation lineage data generated as described in the methods and clustered using the minimum-linkage algorithm of the “bottom-up” method. Panel zero represents the most homologous set of sequences (identical) and panel fifteen represents the least homologous set of sequences (fifteen base substitutions). The darkly colored wedge at the 0° position of each dendritic heat map represents the minimum (red) and maximum (blue) possible heat map relative abundance bin responses of all dendritic heat maps displayed, GC≤50% and GC>50% respectively. White space in the heat maps represents clusters with neutral bin response. Rings, starting at the center, represent clusters of sequences for identity cutoffs of 0.75 to 1.0. Clusters, including single-sequence clusters, are plotted in a radial range that is conserved from the clusters from which they were derived. High resolution versions of all DHMs in this manuscript are available in S2 and S3 Files.

**Fig 4:**
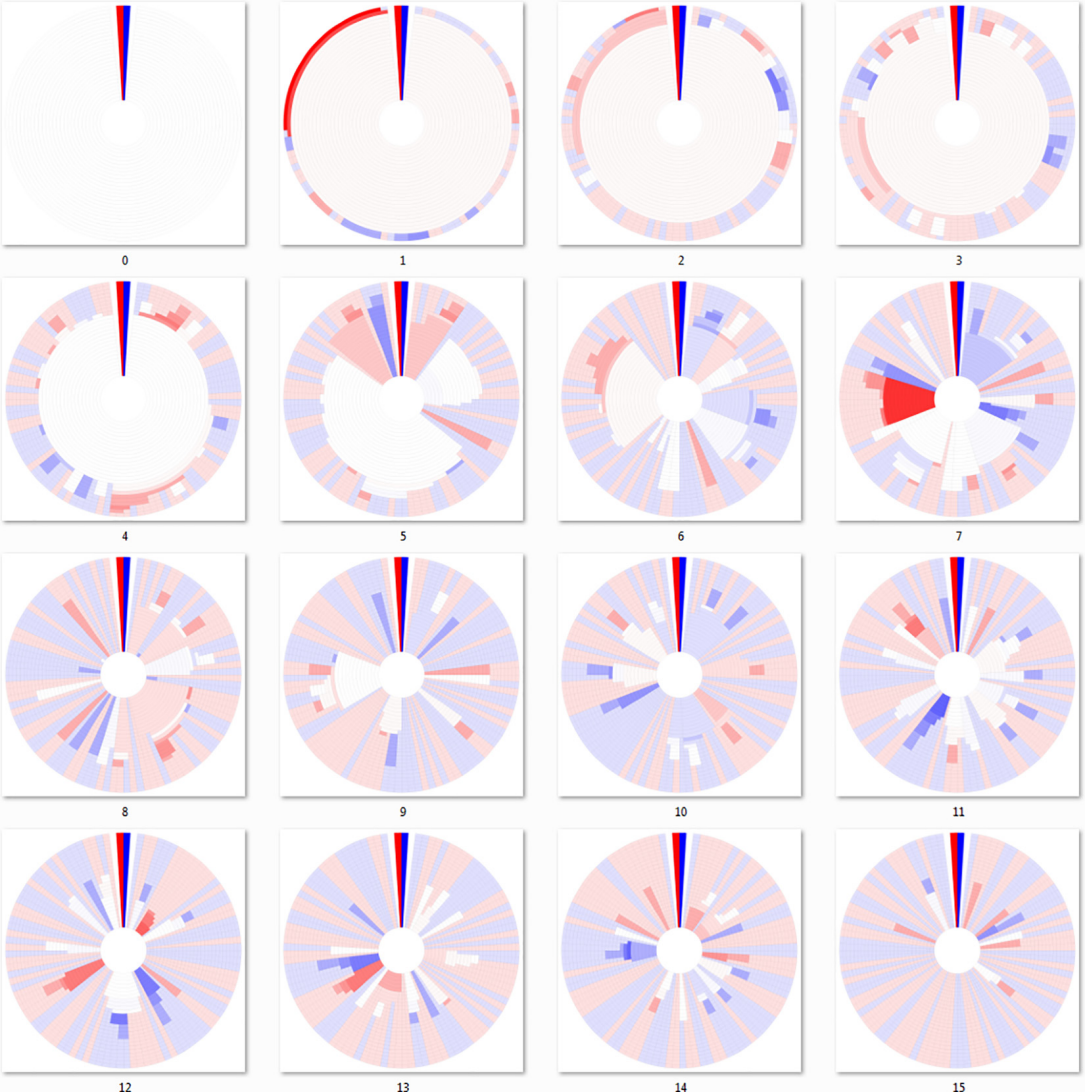
Dendritic heat maps from bottom-up maximum-linkage hierarchical clustering of a mutating population. Dendritic heat maps representing 0 through 15 mutations of the simulated mutation lineage data generated as described in the methods and clustered using the maximum-linkage algorithm of the “bottom-up” method. Panel zero represents the most homologous set of sequences (identical) and panel fifteen represents the least homologous set of sequences (fifteen base substitutions). The darkly colored wedge at the 0° position of each dendritic heat map represents the minimum (red) and maximum (blue) possible heat map relative abundance bin responses of all dendritic heat maps displayed, GC≤50% and GC>50% respectively. White space in the heat maps represents clusters with neutral bin response. Rings, starting at the center, represent clusters of sequences for identity cutoffs of 0.75 to 1.0. Clusters, including single-sequence clusters, are plotted in a radial range that is conserved from the clusters from which they were derived. High resolution versions of all DHMs in this manuscript are available in S2 and S3 Files.

**Fig 5:**
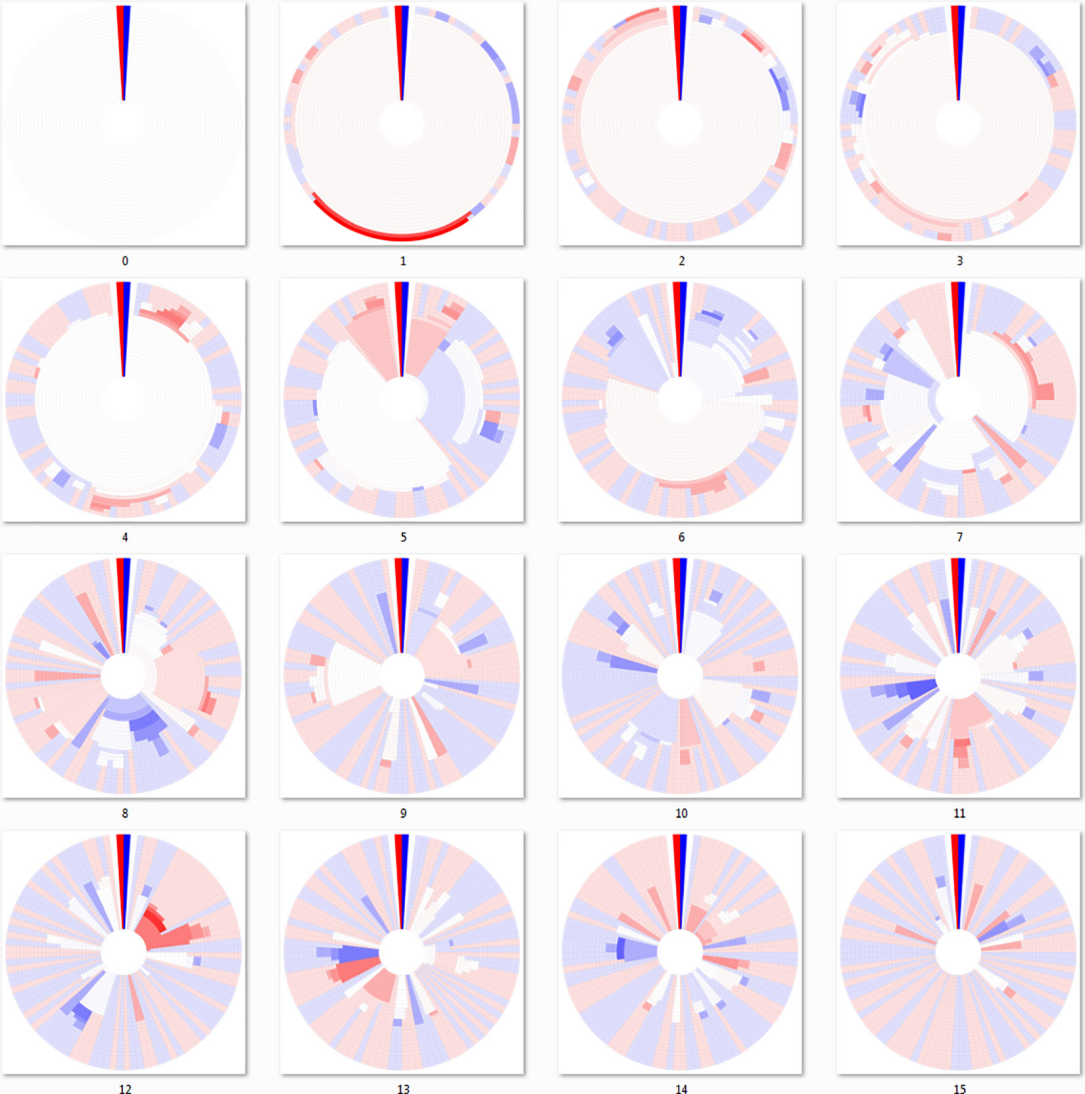
Dendritic heat maps from bottom-up average-linkage hierarchical clustering of a mutating population. Dendritic heat maps representing 0 through 15 mutations of the simulated mutation lineage data generated as described in the methods and clustered using the average-linkage algorithm of the “bottom-up” method. Panel zero represents the most homologous set of sequences (identical) and panel fifteen represents the least homologous set of sequences (fifteen base substitutions). The darkly colored wedge at the 0° position of each dendritic heat map represents the minimum (red) and maximum (blue) possible heat map relative abundance bin responses of all dendritic heat maps displayed, GC≤50% and GC>50% respectively. White space in the heat maps represents clusters with neutral bin response. Rings, starting at the center, represent clusters of sequences for identity cutoffs of 0.75 to 1.0. Clusters, including single-sequence clusters, are plotted in a radial range that is conserved from the clusters from which they were derived. High resolution versions of all DHMs in this manuscript are available in S2 and S3 Files.

There are obvious visible differences between the three linkage algorithms used to cluster the DHMs of Figs 3–5. In all of these “bottom-up” agglomerative algorithms, each cluster starts out as a group of identical sequences or a single sequence. Those clusters of identical sequences are then joined as the clustering cutoff is gradually decreased using the chosen linkage algorithm. As a result, the outermost ring (representing 100% sequence identity) for each respective mutation DHM contains the same clustering breakdown, but possibly in a different configuration due to the ring alignment process. However, the clustering breakdown for the joined clusters of the interior rings is subject to the clustering algorithm and is not the same for each mutation step. For example, the interior rings of the seventh mutation step (panel 7 of Figs 3–5) for each agglomerative algorithm appear different.

The minimum linkage algorithm of Fig 3 produces figures that give the appearance of relatively well-conserved clusters. The transition from a single cluster to multiple clusters occurs at higher identity cutoffs than that of the other algorithms. This apparent overestimation of sequence conservation is expected with the minimum linkage algorithm since clusters are easily joined, only requiring one sequence from each cluster to match one another at the clustering cutoff level. This method has a well-known drawback called the chaining phenomenon, where clusters that have been joined may share only a single close relationship edge while all other edges are very distant [27]. The chaining phenomenon certainly affects the results of Fig 3, especially during the earlier mutations, as it would for many single-linkage DHMs, making single-linkage less than ideal for many data sets.

Fig 4, which was constructed using the maximum-linkage algorithm, contains non-joining cluster segments as mutations progress. While maximum-linkage clustering avoids the chaining phenomenon by requiring all cluster members to have an edge to all other members, it is also an underestimation of sequence conservation and some clusters never join with others for the given cutoff range. This algorithm can be simplified by considering only the furthest edge distance, since all other pairings would be more closely related. In many clusters, a member may be very distantly related to any member of another cluster, regardless of potentially other members in its cluster. This is exactly what can be seen in some mutation iterations in Fig 4, where clusters containing more than one sequence never form edges with all members of another cluster for any given cutoff value, thus never joining.

Like Fig 4, Fig 5 contains many non-joining cluster segments. Using the average-linkage algorithm of Fig 5, clusters are joined if their mean edge distance for all pairs meets the clustering cutoff requirements. The result is similar to the maximum-linkage algorithm because all members of the clusters have an effect on the mean edge distance that dictates if joining will occur. However, average-linkage can be thought of as joining clusters by their “center of cluster mass” instead of a single distant edge, lowering the requirements for joining from that of maximum-linkage and yielding a more accurate apparent sequence conservation.

Due to the chaining phenomenon, minimum-linkage clustering will often be a less than ideal choice for constructing DHMs, although it will still produce a valid DHM. Maximum- and average-linkage algorithms remain viable alternatives, however their propensity to form non-joining cluster segments and the underestimation of sequence conservation by maximum-linkage also makes them less than ideal. In terms of information gained, it is not likely that a non-joining cluster segment does much to aid in visually comparing how deeply rooted a phenotype is across multiple genotypes. Like dendrogram building in general, the algorithm used often comes down to user preference. However, the need to construct a distance matrix, a step that can require large amounts of computer memory and time, and a propensity for creating non-joining cluster segments makes all three “bottom-up” approaches less than ideal for large data sets.

### 3.3 Top-down Hierarchical Clustering

**Fig 6:**
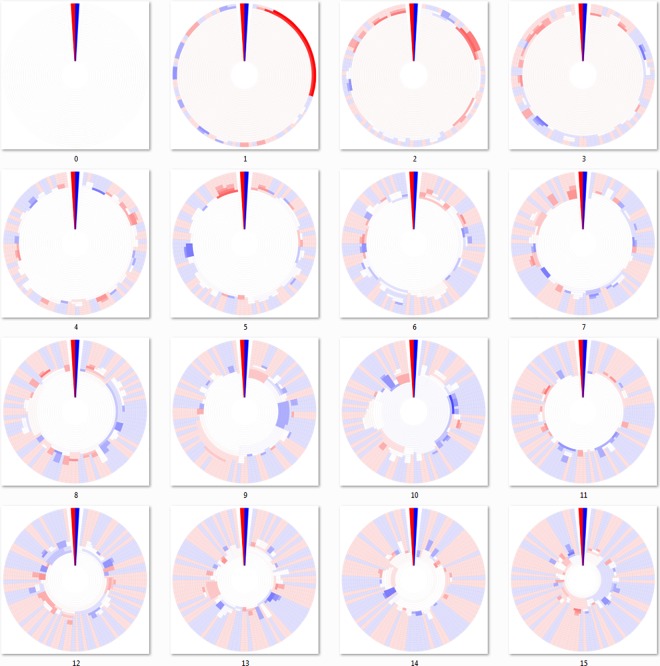
Dendritic heat maps from top-down hierarchical clustering of a mutating population. Dendritic heat maps representing 0 through 15 mutations of the simulated mutation lineage data generated as described in the methods and clustered using the “top-down” method. Panel zero represents the most homologous set of sequences (identical) and panel fifteen represents the least homologous set of sequences (fifteen base substitutions). The darkly colored wedge at the 0° position of each dendritic heat map represents the minimum (red) and maximum (blue) possible heat map relative abundance bin responses of all dendritic heat maps displayed, GC≤50% and GC>50% respectively. White space in the heat maps represents clusters with neutral bin response. Rings, starting at the center, represent clusters of sequences for identity cutoffs of 0.75 to 1.0. Clusters, including single-sequence clusters, are plotted in a radial range that is conserved from the clusters from which they were derived. High resolution versions of all DHMs in this manuscript are available in S2 and S3 Files.

The algorithms of the “bottom-up” approaches require constructing a distance matrix from pairwise identity or similarity calculations between all sequences, which for large datasets can lead to impractically large memory or computational time requirements. For this reason, we implemented a “top-down” hierarchical clustering method that splits clusters by incrementally increasing the sequence identity required to bin sequences, similar to previously described divisive methods [26,28]. The “top-down” approach does not require a large comprehensive distance matrix to be built and uses centroid-based clustering, where clusters are split if multiple cluster “centroid” elements can be separated with the given clustering cutoff. Centroids are then used as a database for a sequence search algorithm to assign closest matching sequences or new centroids if the closest match is out of the cutoff range [1]. The dendritic heat maps of Fig 6 were constructed using a top-down approach. A brief example of the top-down clustering is available in S1 File.

The most fundamental difference between the “top-down” and all three “bottom-up” algorithms is that the DHM is created by splitting clusters, rather than joining them. The figure is constructed by first assigning sequences to clusters at the lowest cutoff level, which corresponds to the innermost ring on all DHMs, then splitting those clusters for subsequent DHM rings as the clustering cutoff is incrementally increased.

The advantages of the “top-down” centroid-based clustering approach over “bottom-up” approaches are speed, memory requirements, and a more intuitive view of sequence conservation as data size increases. Circumventing the construction of a distance matrix has obvious advantages in speed and memory requirements for sufficiently large data sets, where a distance matrix becomes impractically large. The apparent sequence conservation of the “top-down” DHM is more intuitive in that it avoids the chaining phenomenon of single-linkage and also avoids the non-joining cluster segments of maximum- and average-linkage, potentially leading to more informative observations of the level at which genotype divergence (cluster branching) has an effect on phenotype divergence (relative abundance visualized via differing heat map bin response). The “top-down” approach has a slight bias away from forming non-joining cluster segments because clusters must pass a threshold to be split, rather than having to pass a threshold to be joined as is the case with “bottom-up” approaches.

In all cases, including the three “bottom-up” approaches, sequence length and homology can have significant effects on the clustering layout of DHMs. Shorter sequences and also less homologous sequences would result in more non-joining clusters, while the opposite conditions would result in more cluster joining. In a study that tracks sequence homology of different lineages, as is simulated in our mutation data set, non-joining cluster segments would appear sooner in data sets with shorter sequences and faster mutation rates. Therefore, there are potential data conditions where even the “top-down” approach yields less-informative DHMs.

Summary tables of the sequence and cluster counts from the mutation dataset DHMs are provided as an Excel document in S3 File, showing different bin distributions for each of the algorithms which affects their appearance. As previously described with the DHM appearances, the tables reiterate that the minimum-linkage algorithm bins sequences together more readily than the others, while the maximum-linkage and average-linkage algorithms are more exclusive. The centroid-based algorithm, which does not use a distance matrix to determine cluster similarity, occupies a binning inclusiveness middle ground between the three others. It is our opinion that the moderate inclusiveness of centroid-based clustering, as well as its ability to cluster larger datasets (described in more detail in the following section), makes it the best option of the four to construct DHMs. However, just as each of those clustering algorithms is a valid technique, each of the DHMs that are constructed from their clusters is also valid.

### 3.4 Dendritic Heat Maps of a Growing Population

**Fig 7:**
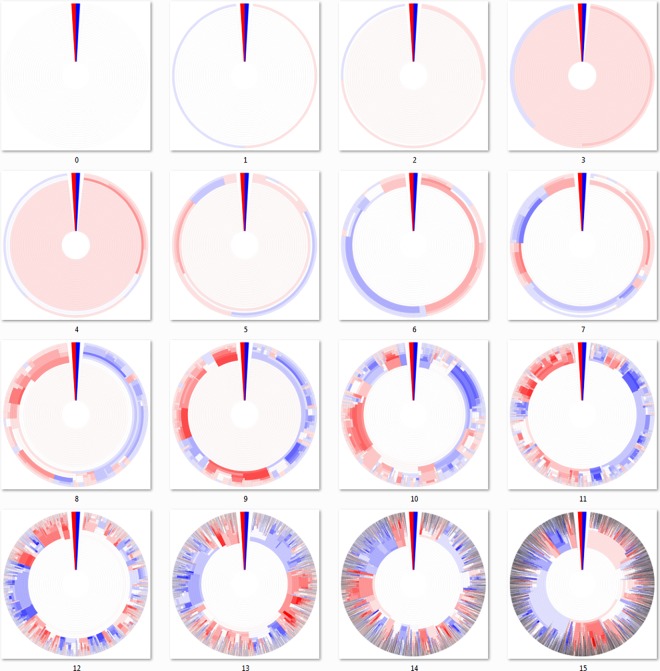
Dendritic heat maps from top-down hierarchical clustering of a growing population. Dendritic heat maps representing generations 0 through 15 of the simulated population growth data generated as described in the methods and clustered using the “top-down” method. The darkly colored wedge at the 0° position of each dendritic heat map represents the minimum (red) and maximum (blue) possible heat map relative abundance bin responses of all dendritic heat maps displayed, GC ≤50% and GC>50% respectively. White space represents clusters with neutral bin response. Rings, starting at the center, represent clusters of sequences for identity cutoffs of 0.75 to 1.0. Clusters, including single-sequence clusters, are plotted in a radial range that is conserved from the clusters from which they were derived. High resolution versions of all DHMs in this manuscript are available in S2 and S3 Files.

DHMs can be scaled to fit a wide range of dataset sizes while maintaining their dendrogram layout. Fig 7 shows DHMs of the artificially generated dataset that simplifies mutation and growth as substitutions are introduced without fatal consequences into generations, displaying an increasing complexity (as described in the methods section as the population growth data set). Up to fifteen generations, including the initial sequence at generation 0, are shown as individual DHMs. The number of total sequences doubles for each generation, increasing data size and complexity exponentially. This dataset is used to show the scalability of DHMs.

The DHM for generation 0 represents the simplest possible cluster configuration, displaying information for only a single sequence. For a fixed sequence length as in the artificial data used here, there is a theoretical final cluster distribution, where mutations have progressed to a point where additional sequences can no longer be unique. The cluster distribution for the DHM of this theoretical endpoint would appear symmetrical, and all evolutionary paths end at this same fixed endpoint cluster distribution. The number of unique sequences in the endpoint cluster distribution is calculable at *b*^*n*^, where *b* is the number of possible base choices and *n* is the total number of bases used in the simulated DNA sequences. The generations that are displayed in Fig 7 are snapshots into one of the many pathways toward the theoretical endpoint cluster distribution of *b*^*n*^ unique sequences. This theoretical endpoint holds true for traditional dendrograms as well, however DHMs have the added dimension of displaying relative abundance information (heat map bin response). The random qualities built into our sequence generation with the added dimension of heat map distribution yields many DHM colorations for the fixed endpoint cluster distribution discussed above.

Drawing parallels to natural data, for every set of samples, there is also a theoretical fixed endpoint cluster distribution. While natural data does not have fixed sequence lengths, there is likely a range of lengths that are allowed by evolutionary pressures and there would be many possible evolutionary paths toward the endpoint cluster distribution [29,30]. However, the difference with a natural data set is that generations are determined by evolutionary processes that are much more complex than random non-lethal base substitutions [31,32]. Essentially, the artificial data set endpoint would display every possible lineage while natural data would have fatal dendrogram branches trimmed out of the endpoint cluster distribution, likely in a non-symmetrical distribution. The complexity that is common in natural samples would likely yield many possible DHM colorations, similar to an artificial data set. For these reasons, it is not unreasonable to use artificially-generated data to show DHMs of population growth.

Fig 7 is an informative way to introduce the evolutionary context of DHMs, where each displays a multi-level snapshot into the phenotype history of a sample. Each snapshot represents a view from the same evolutionary path, one of the many paths toward the theoretical endpoint. As each generation doubles in size, in many cases it is easy to visually track the growth and divergence of individual clusters from generation to generation. As new unique sequences are added, new clusters in the outermost ring are created and inner clusters diverge to account for their addition. Likewise, when duplicate sequences are added, their respective sections increase in width, which represents cluster size. Phenotype divergences occur deeper into the DHMs as generations progress and population genotypes diverge. Eventually, we are able to see increasing fracturing of genotypes and phenotypes as the total population becomes more complex. While something similar to Fig 7 could be recreated with experimental evolution datasets, a natural dataset would yield only a single DHM unless a time component is involved. However, even with a time element involved, it is unlikely to find a natural sample as simple as the earliest generations of Fig 7.

### 3.5 Application

**Fig 8:**
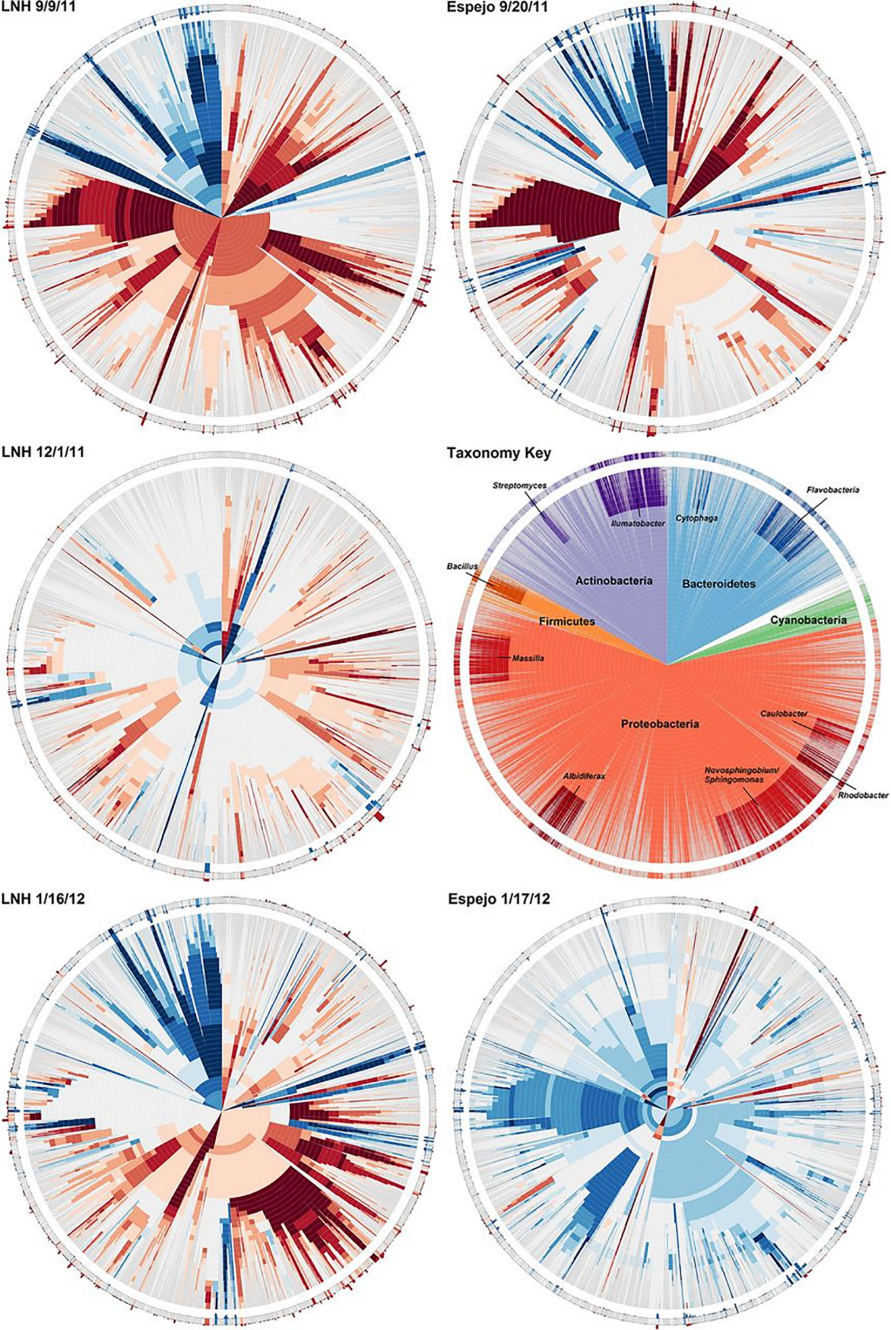
Fig 3 reused from Elser *et al*. 2014 with the kind permission of ASM. Dendritic heat maps displaying habitat preferences for multiple levels of phylogenetic clades across multiple time points and locations. Reprinted from [33] under a CC BY license, with permission from AEM, original copyright 2014.

**Fig 9:**
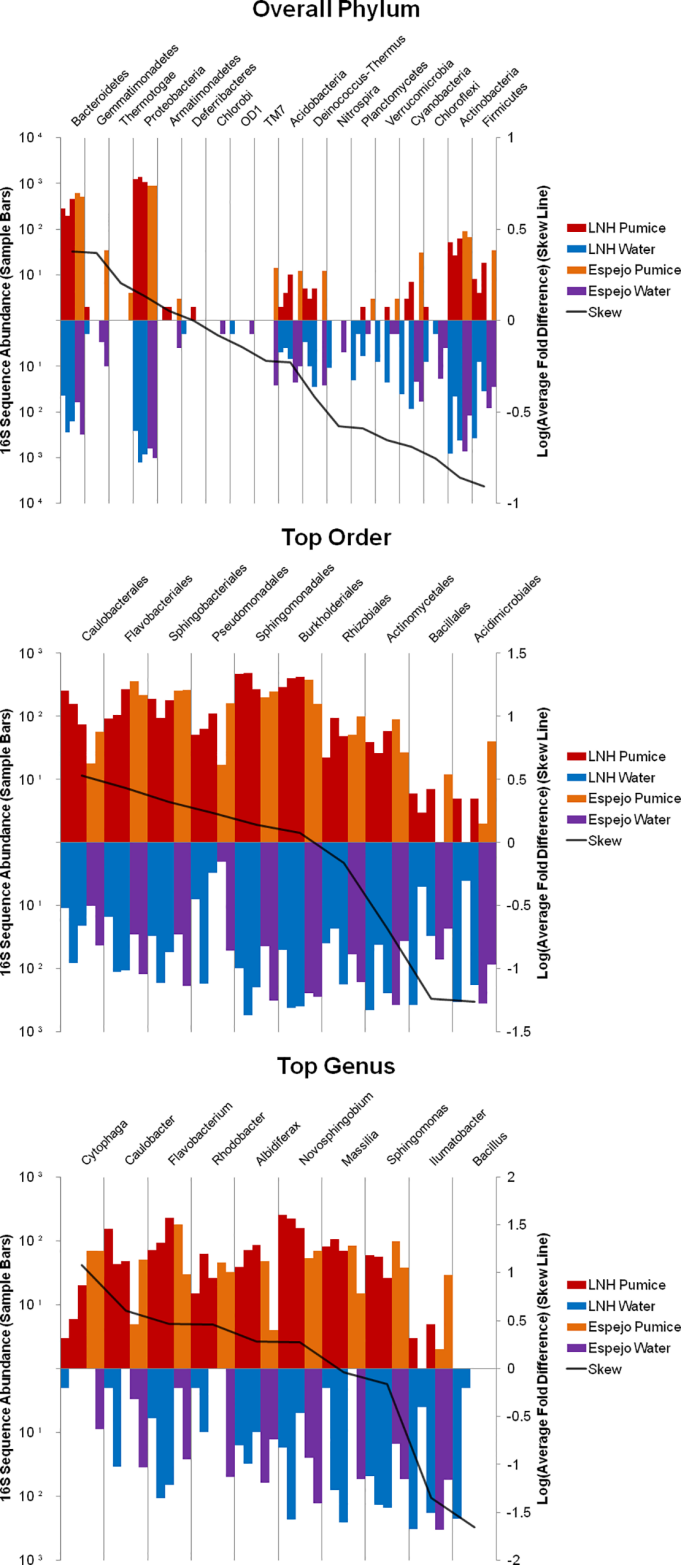
Fig 4 reused from Elser *et al*. 2014 with the kind permission of ASM. Histograms displaying the strongest habitat preferences for the phylum, order, and genus taxonomical levels of four sample types. A skew line is used to show the relative strength of habitat preference. Reprinted from [33] under a CC BY license, with permission from AEM, original copyright 2014.

Recently, DHMs were used to describe large and complex microbial community sequence data from aquatic pumice samples, where the goal was to show microbial habitat preference at multiple levels of taxonomical classification [33]. While seemingly a straightforward task of counting homologous sequences for each habitat, the problem of similarity cutoff choice can influence how sequences are binned and ultimately expressed in a heat map. In Fig 8 (Fig 3 of Elser *et al*. [33]) (original copyright 2014, Applied and Environmental Microbiology), sequences maintain the same radial position throughout each of the DHMs. For nearly all sequences, the strength, and sometimes direction, of their heat map expression changes depending on the specificity of the clustering cutoff used to bin them. Fig 9 (Fig 4 of Elser *et al*. [33]) (original copyright 2014, Applied and Environmental Microbiology) shows histograms of the same data being binned according to Ribosomal Database Project taxonomic classifications [33]. Essentially, these histogram bar heights translate to heat map color intensity and convey the same information in different formats. Of course, binning based on taxonomic classification does not exactly represent binning based on sequence identity, but it can be a close and familiar approximation. The issue with clustering cutoff choice is perfectly represented in Fig 9 (Fig 4 of Elser *et al*. [33]), where depending on the level at which sequences are binned, the scale of the “Skew Line” axis changes to accommodate the range in relative abundance bin response, which translates to heat map color and intensity. DHMs on the other hand embrace this effect of clustering specificity on binning and bin response, where it is used to show the effect of homology on heat map response.

**Fig 10:**
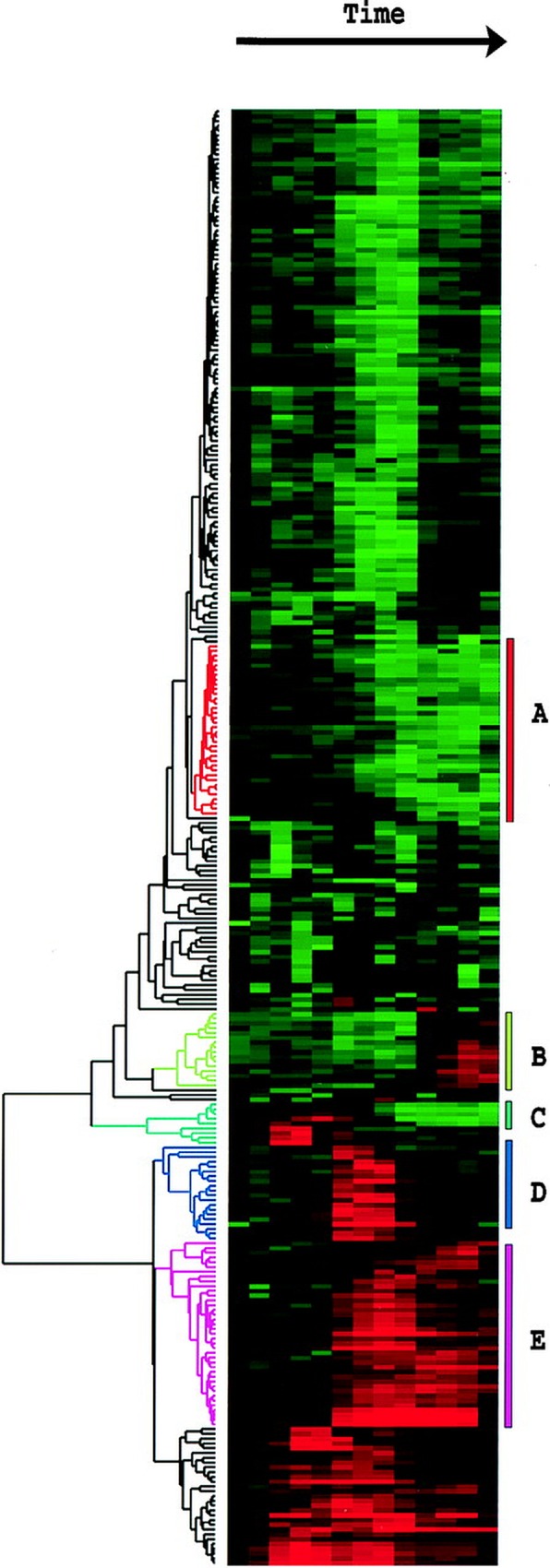
Fig 1 reused from Eisen *et al*. 1998 with the kind permission of PNAS. Heat map displaying data from a time course of serum stimulation of primary human fibroblasts. Reprinted from [34] under a CC BY license, with permission from PNAS, original copyright 1998.

In a publication by Eisen *et al*., heat maps are used to describe *Saccharomyces cerevisiae* genome microarray data for a series of time points [34]. Each row in Fig 10 (Eisen *et al*. [34] Fig 1) (original copyright 1998, The National Academy of Sciences) represents individual genes, which in terms of binning are sequences at 100% identity or a fractional identity clustering cutoff of 1.0, and each column represents a time point. Fig 10 (Fig 1 of Eisen *et al*. [34]) shows rows being clustered and arranged based on their heat map response and a dendrogram is provided to display the cladistics of row bin responses, not row sequence identity, which is useful for displaying clades of similar patterns of expression. However, the goal of DHMs is to display the effect of sequence homology and genotype clades with phenotype heat map bin responses. Two important differences between the DHMs introduced in this work and many published traditional heat maps, including those in Eisen *et al*., are the inclusion of multiple heat maps for a single sample (represented by a column in Eisen *et al*. [34]) and sequence identity rather than heat map expression pattern determining row arrangement (or radial position in the case of radial heat maps). If we were to convert the work of Eisen *et al*. into DHMs, each column of Fig 10 (Fig 1 of Eisen *et al*. [34]) would have their own DHM with multiple levels of clustering arranged by sequence identity so that we could see how well the heat map expression pattern for a gene is conserved among its homologs. It would be possible to create a separate cladogram that represents bin response pattern similarity (or dissimilarity) using Bray-Curtis dissimilarity, however this is beyond the scope of the work presented here [35].

## 4.0 Conclusion

DHMs represent a powerful tool for visualizing correlations in genotype and phenotype changes across evolutionary space and time, and will ultimately help decipher dynamic processes in complex, natural communities such as metatranscriptomes, where similarities occur across a multitude of scales. The “top-down” approach that we outline here provides an efficient method of constructing DHMs that display phenotype relative abundance divergence with homology divergence and is the method that we recommend for most cases, however, any hierarchical clustering method can be used for DHM construction. While this paper discusses the application of DHMs in an exclusively nucleic acid sequence context, their range is certainly not limited to sequence information and can be used in any dataset that has a pair of groups that share underlying traits.

## Supporting Information

S1 File DHM construction and top-down algorithm. This file contains a brief example of the dendritic heat map construction process as well as the top-down algorithm.
(PDF)

S2 File ZIP file containing Perl scripts, images, and configuration files for the bottom-up approach. This file is a zipped folder containing the bottom-up Perl script described in the methods section, high-resolution versions of the images in the discussion, and the configuration files used by Circos to plot the images.
(ZIP)

S3 File ZIP file containing Perl scripts, images, and configuration files for the top-down approach. This file is a zipped folder containing the top-down Perl scripts described in the methods section, high-resolution versions of the images in the discussion, the configuration files used by Circos to plot the images, FASTA files of the artificial datasets, and an Excel file summary of the clusters for each mutation dataset DHM.
(ZIP)
