# Supplementary material for: Using Dendritic Heat Maps to Simultaneously Display Genotype Divergence with Phenotype Divergence
Source: PLoS One. 2016 Aug 18;11(8):e0161292. doi: 10.1371/journal.pone.0161292 (PMC4990276; doi:10.1371/journal.pone.0161292)
Supplement: S1 File — This file contains a brief example of the dendritic heat map construction process as well as the top-down algorithm. (PDF) [file pone.0161292.s001.pdf]

Dendritic Heat Map Construction

In the following document, we will discuss the construction of dendritic heat maps (DHMs) for the bottom-up and top-down approaches. This document does not show actual clustering, all clusters within are hypothetical and designed for demonstrational purposes. DHM construction is not a clustering methodology, it is a downstream visualization of already hierarchical clustered data. However, the top-down approach does require manual manipulation of the sequence input order due to the use of centroid-based clustering. The first half of this document focuses on DHM construction which can typically be done directly following bottom-up clustering methods. The second half of this document focuses on setting up data sets to use centroid-based clustering (not performed here) to create top-down hierarchical clusters over a range of clustering cutoffs that can then be used to create a DHM. For both approaches, we are ultimately building a set of input files that are readable by the Perl package Circos, which plots the DHM image.

The bottom-up agglomerative clustering algorithms all follow a process of comparing sequences and joining clusters as the clustering cutoff is decreased from 1.0 to the specified cutoff. To construct the dendritic heat maps from these clustering steps, we used the containing sequences and their counts for each ring. To illustrate the construction of a dendritic heat map here, we will use a small hypothetical dataset of ten sequences (A-J). We will assign these sequences to two groups, consonants and vowels, which will be used to assign color to the DHM clusters.

To begin, we will perform a multiple alignment of the ten sequences to acquire a tree-output ordering. In our work we used Clustal Omega, however any multiple alignment program that outputs a tree-ordering can be used. Likewise, we used USEARCH for both the agglomerative clustering and centroid-based clustering but any clustering program can be used as long as the DHM construction procedures remain the same.

**Unaligned Sequences**	**Aligned Sequences**
Sequence A	Sequence D
Sequence B	Sequence J
Sequence C	Sequence A
Sequence D	Sequence B
Sequence E	Sequence I
Sequence F	Sequence H
Sequence G	Sequence C
Sequence H	Sequence E
Sequence I	Sequence G
Sequence J	Sequence F

Once we have obtained our tree-ordered multiple alignment, we can use this order as a starting point to place clusters onto the dendritic heat map. In our small hypothetical example, we will be creating a dendritic heat map that covers a clustering cutoff range of 0.95-1.0 for these ten sequences. DHM construction is started at the innermost ring, regardless of the

clustering approach used. In our example, all of the sequences will cluster together into a single cluster at the initial cutoff of 0.95.

0.95

Clustering Cutoff:	0.95
Cluster:	Cluster 1
Sequences:	Sequence D
	Sequence J
	Sequence A
	Sequence B
	Sequence I
	Sequence H
	Sequence C
	Sequence E
	Sequence G
	Sequence F

The ordering placement of this single cluster on the first ring is inconsequential, but we will nonetheless proceed with the steps to do so. These steps are important since clustering programs and multiple alignment programs do not always yield the same neighboring sequence relationships. To ensure that the cluster relationships remain intact and are still arranged in a meaningful configuration, we use a combination of multiple alignment ordering and clustering relationships. We will first look to see if the cluster contains the first sequence of the multiple alignment ordering. Since this cluster does contain the first sequence, we "place" this cluster on to the DHM, noting the number of sequences contained within the cluster (ten). We then order the sequences within the "placed" cluster according to the multiple alignment, looking for the first sequence and bringing it to the top of the cluster if found, and so on until reaching the end of the sequences within the cluster.

The final step for this ring is to assign a color value to the cluster. The heat map hues are partitioned to have gradual changes with twenty-three possible categories (two sequential 11-color Brewer palettes and white) and is determined by the logarithmic value of the ratio of sequences from each group, log((Vowels+1)/(Consonants+1)). The Circos package will determine which color to assign to the log value given, based on normalizing the range of log values in the file and the colors that are provided to it. Red hue indicates relative abundance bin average toward the vowel group (≤50%) and blue hue indicates relative abundance bin average toward consonant group (>50%), with white being neutral. Hue luminosity corresponds to the strength of the heat map response. In addition, we assign small markers at the 0° position of the DHM to indicate the minimum and the absolute value maximum of the color hues for the entire DHM. Our color value calculation for this cluster is:

$$\text{Log}((3+1)/(7+1)) \approx -0.301$$

Our Circos input file for this 0.95 ring would include the radial position where this cluster started and stopped, as well as min/max hue markers:

DHM 2 12 -0.301
DHM 0 1 -0.301
DHM 13 14 0.301

Summary:

**DHM name**	**Start Position**	**Stop Position**	**Color**
DHM	2	12	-0.301
DHM	0	1	-0.301
DHM	13	14	0.301

The output of the DHM thus far will have a single ring representing the clustering cutoff of 0.95, consisting of a single ring:

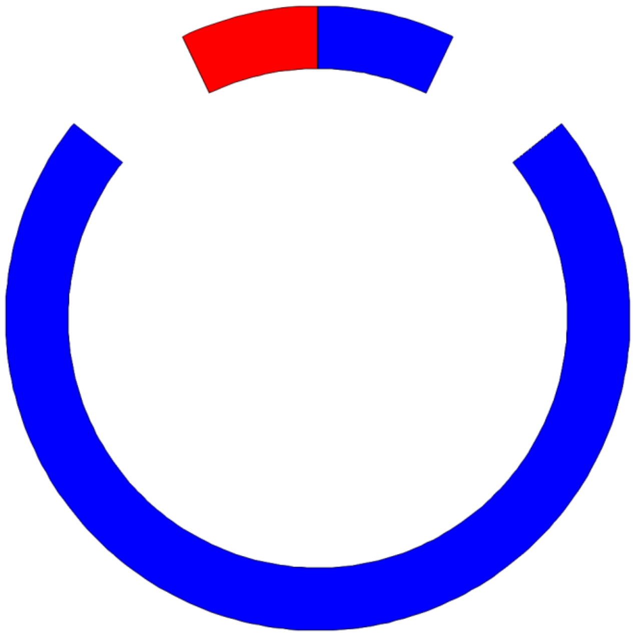

If this first ring had more than one cluster, we would continue with this process until completing the ring, as you will see in the next ring. Since this ring is finished, we output the sequence order of all clusters on this ring to be used as the input order of the next ring (instead of the multiple alignment order). This step is crucial to conserving the radial position of each sequence throughout all rings. The output order of this 0.95 ring, which will serve as the input order of the 0.96 ring is. We will call this the "Current Alignment Order":

Sequence D
Sequence J
Sequence A
Sequence B
Sequence I
Sequence H
Sequence C
Sequence E
Sequence G
Sequence F

Now that the initial 0.95 ring is complete, we move on to the 0.96 cutoff clustering files that will be used to make the next ring. In our hypothetical clustering run, a 0.96 cutoff yielded two clusters:

0.96

Clustering Cutoff:	0.96
Cluster:	Cluster 1
Sequences:	Sequence D
	Sequence A
	Sequence I
	Sequence F
Cluster:	Cluster 2
Sequences:	Sequence J
	Sequence B
	Sequence H
	Sequence C
	Sequence E
	Sequence G

Our first step with these clusters is to begin with the first sequence in the Current Alignment Order, Sequence D. Cluster 1 contains Sequence D so we begin by “placing” it on to the ring and ordering the sequences contained within it. We continue down the Current Alignment Order and place cluster 2. Following the Current Alignment Order, the order of sequences within cluster 1 and 2 will be:

**Cluster 1**	**Cluster 2**
Sequence D	Sequence J
Sequence A	Sequence B
Sequence I	Sequence H
Sequence F	Sequence C
	Sequence E
	Sequence G

The output order of this 0.96 ring, which will serve as the input order of the 0.97 ring is:

Sequence D
Sequence A
Sequence I
Sequence F
Sequence J
Sequence B
Sequence H
Sequence C
Sequence E
Sequence G

After calculating the color log values for each cluster, our Circos input file for this 0.96 ring would look like:

DHM 2 6 0
DHM 6 12 -0.477
DHM 0 1 -0.477
DHM 13 14 0.477

Summary:

DHM name	Start Position	Stop Position	Color
DHM	2	6	0
DHM	6	12	-0.477
DHM	0	1	-0.477
DHM	13	14	0.477

The output of the DHM thus far will have two rings representing the clustering cutoffs of 0.95 and 0.96. Since the absolute minimum and maximum color values thus far are -0.477 and 0.477, respectively, the marker values for each ring are edited to those values:

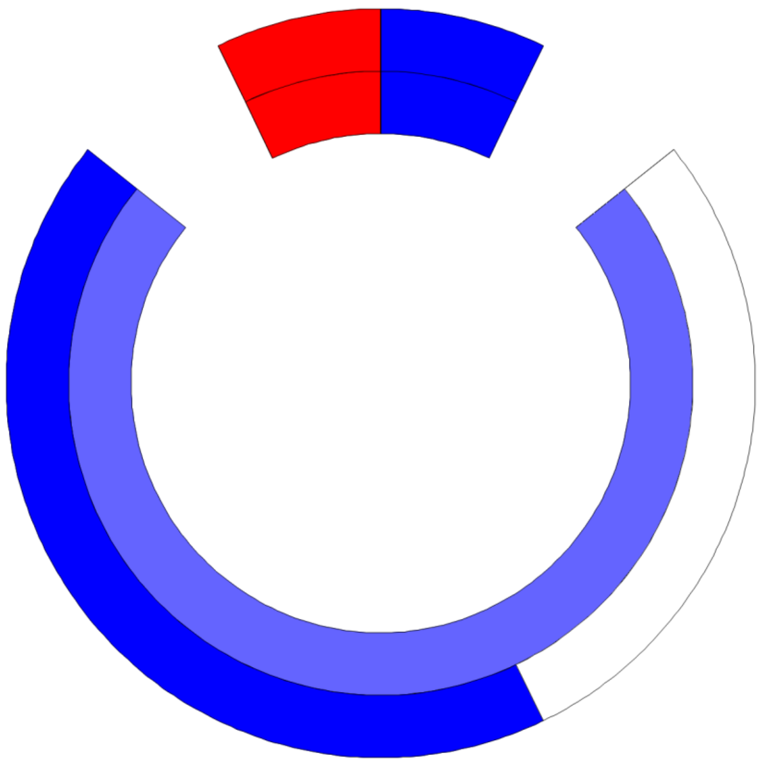

0.97

Following the same process of placing clusters and then ordering them and using the result as the order for the next ring, we will fill out the rest of the DHM.

Clustering Cutoff:	0.97
Cluster:	Cluster 1
Sequences:	Sequence D
Cluster:	Cluster 2
Sequences:	Sequence A
	Sequence I
	Sequence F
Cluster:	Cluster 3
Sequences:	Sequence J
	Sequence B
	Sequence G
Cluster:	Cluster 4
Sequences:	Sequence C
	Sequence E
	Sequence H

After ordering these clusters by the output order of the previous ring:

**Cluster 1**	**Cluster 2**	**Cluster 3**	**Cluster 4**
Sequence D	Sequence A	Sequence J	Sequence H
	Sequence I	Sequence B	Sequence C
	Sequence F	Sequence G	Sequence E

The output order of this 0.97 ring, which will serve as the input order of the 0.98 ring is:

Sequence D
Sequence A
Sequence I
Sequence F
Sequence J
Sequence B
Sequence G
Sequence H
Sequence C
Sequence E

After calculating the color log values for each cluster, our Circos input file for this 0.97 ring would look like:

DHM 2 3 -0.301
DHM 3 6 0.176
DHM 6 9 -0.602
DHM 9 12 -0.176
DHM 0 1 -0.602
DHM 13 14 0.602

Summary:

DHM Name	Start Position	Stop Position	Color
DHM	2	3	-0.301
DHM	3	6	0.176
DHM	6	9	-0.602
DHM	9	12	-0.176
DHM	0	1	-0.602
DHM	13	14	0.602

The output of the DHM thus far will have three rings representing the clustering cutoffs of 0.95 - 0.97. Since the absolute minimum and maximum color values thus far are -0.602 and 0.602, respectively, the marker values for each ring are edited to those values:

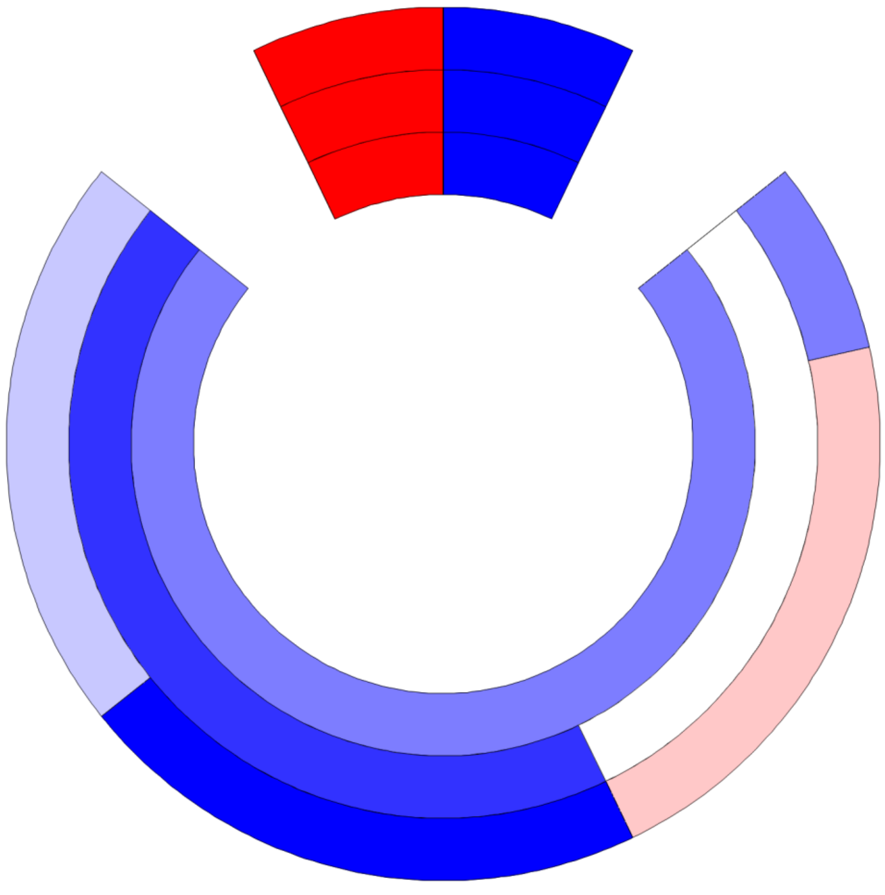

0.98

Clustering Cutoff:	0.98
Cluster:	Cluster 1
Sequences:	Sequence D
Cluster:	Cluster 2
Sequences:	Sequence A
	Sequence I
Cluster:	Cluster 3
Sequences:	Sequence F
Cluster:	Cluster 4
Sequences:	Sequence J
	Sequence G
Cluster:	Cluster 5
Sequences:	Sequence B
Cluster:	Cluster 6
Sequences:	Sequence H
	Sequence C
	Sequence E

**Cluster 1**	**Cluster 2**	**Cluster 3**	**Cluster 4**	**Cluster 5**	**Cluster 6**
Sequence D	Sequence A	Sequence F	Sequence J	Sequence B	Sequence C
	Sequence I		Sequence G		Sequence E
					Sequence H

The output order of this 0.98 ring, which will serve as the input order of the 0.99 ring is:

Sequence D
Sequence A
Sequence I
Sequence F
Sequence J
Sequence G
Sequence B
Sequence H
Sequence C
Sequence E

After calculating the color log values for each cluster, our Circos input file for this 0.98 ring would look like:

DHM 2 3 -0.301
DHM 3 5 0.477
DHM 5 6 -0.301
DHM 6 8 -0.477
DHM 8 9 -0.301
DHM 9 12 -0.176
DHM 0 1 -0.602
DHM 13 14 0.602

Summary:

DHM Name	Start Position	Stop Position	Color
DHM	2	3	-0.301
DHM	3	5	0.477
DHM	5	6	-0.301
DHM	6	8	-0.477
DHM	8	9	-0.301
DHM	9	12	-0.176
DHM	0	1	-0.602
DHM	13	14	0.602

The output of the DHM thus far will have four rings representing the clustering cutoffs of 0.95 - 0.98. Since the absolute minimum and maximum color values thus far are -0.602 and 0.602, respectively, the marker values for each ring are edited to those values:

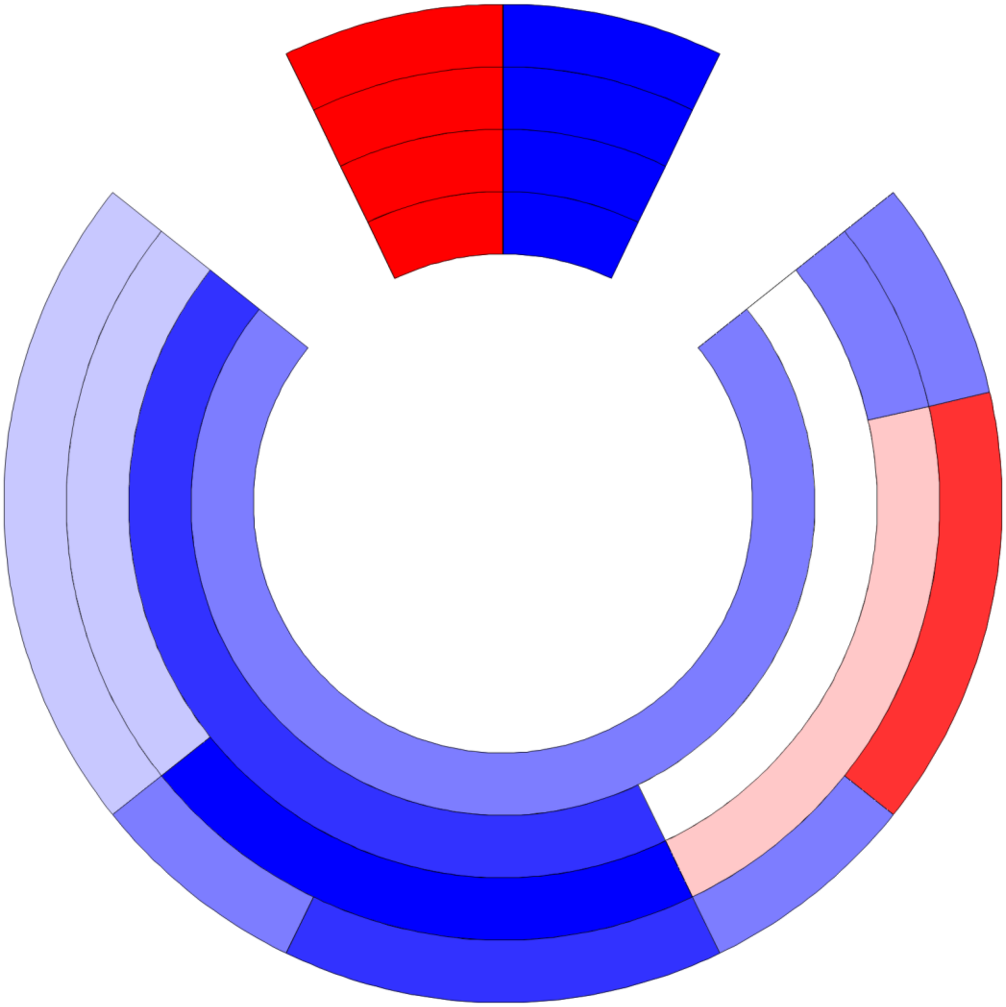

0.99

Clustering Cutoff:	0.99
Cluster:	Cluster 1
Sequences:	Sequence D
Cluster:	Cluster 2
Sequences:	Sequence A<br>Sequence I
Cluster:	Cluster 3
Sequences:	Sequence F
Cluster:	Cluster 4
Sequences:	Sequence J
Cluster:	Cluster 5
Sequences:	Sequence G
Cluster:	Cluster 6
Sequences:	Sequence B
Cluster:	Cluster 7
Sequences:	Sequence H<br>Sequence C<br>Sequence E

**Cluster 1**	**Cluster 2**	**Cluster 3**	**Cluster 4**	**Cluster 5**	**Cluster 6**
Sequence D	Sequence A<br>Sequence I	Sequence F	Sequence J	Sequence G	Sequence B

**Cluster 7**
Sequence H<br>Sequence C<br>Sequence E

The output order of this 0.99 ring, which will serve as the input order of the 1.0 ring is:

Sequence D
Sequence A
Sequence I
Sequence F
Sequence J
Sequence G
Sequence B
Sequence H
Sequence C
Sequence E

After calculating the color log values for each cluster, our Circos input file for this 0.99 ring would look like:

DHM 2 3 -0.301
DHM 3 5 0.477
DHM 5 6 -0.301
DHM 6 7 -0.301
DHM 7 8 -0.301
DHM 8 9 -0.301
DHM 9 12 -0.176
DHM 0 1 -0.602
DHM 13 14 0.602

Summary:

**DHM Name**	**Start Position**	**Stop Position**	**Color**
DHM	2	3	-0.301
DHM	3	5	0.477
DHM	5	6	-0.301
DHM	6	7	-0.301
DHM	7	8	-0.301
DHM	8	9	-0.301
DHM	9	12	-0.176
DHM	0	1	-0.602
DHM	13	14	0.602

The output of the DHM thus far will have five rings representing the clustering cutoffs of 0.95 - 0.99 Since the absolute minimum and maximum color values thus far are -0.602 and 0.602, respectively, the marker values for each ring are edited to those values:

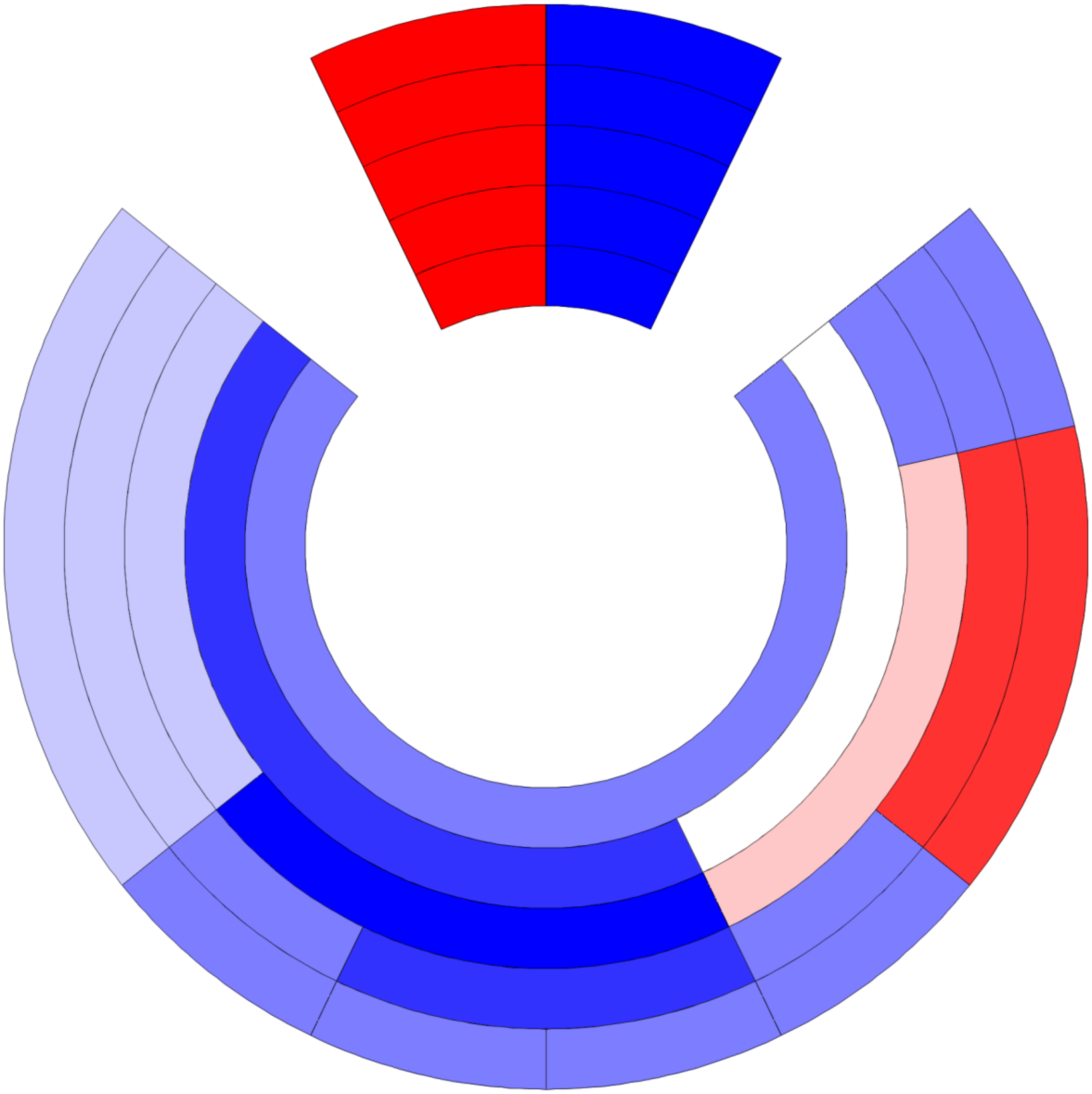

1.0

Clustering Cutoff:	1.0
Cluster:	Cluster 1
Sequences:	Sequence D
Cluster:	Cluster 2
Sequences:	Sequence A
Cluster:	Cluster 3
Sequences:	Sequence I
Cluster:	Cluster 4
Sequences:	Sequence F
Cluster:	Cluster 5
Sequences:	Sequence J
Cluster:	Cluster 6
Sequences:	Sequence G
Cluster:	Cluster 7
Sequences:	Sequence B
Cluster:	Cluster 8
Sequences:	Sequence E
Cluster:	Cluster 9
Sequences:	Sequence C<br>Sequence H

**Cluster 1**	**Cluster 2**	**Cluster 3**	**Cluster 4**	**Cluster 5**	**Cluster 6**
Sequence D	Sequence A	Sequence I	Sequence F	Sequence J	Sequence G
**Cluster 7**	**Cluster 8**	**Cluster 9**			
Sequence B	Sequence E	Sequence C<br>Sequence H			

The output order of this 1.0 ring, which will be the final order is:

Sequence D
Sequence A
Sequence I
Sequence F
Sequence J
Sequence G
Sequence B
Sequence E
Sequence C
Sequence H

After calculating the color log values for each cluster, our Circos input file for this 1.0 ring would look like:

DHM 2 3 -0.301
DHM 3 4 0.301
DHM 4 5 0.301
DHM 5 6 -0.301
DHM 6 7 -0.301
DHM 7 8 -0.301
DHM 8 9 -0.301
DHM 9 10 0.301
DHM 10 12 -0.477
DHM 0 1 -0.602
DHM 13 14 0.602

Summary:

**DHM Name**	**Start Position**	**Stop Position**	**Color**
DHM	2	3	-0.301
DHM	3	4	0.301
DHM	4	5	0.301
DHM	5	6	-0.301
DHM	6	7	-0.301
DHM	7	8	-0.301
DHM	8	9	-0.301
DHM	9	10	0.301
DHM	10	12	-0.477
DHM	0	1	-0.602
DHM	13	14	0.602

The output of the DHM thus far will have six rings representing the clustering cutoffs of 0.95 – 1.0. Since the absolute minimum and maximum color values are -0.602 and 0.602, respectively, the marker values for each ring are edited to those values:

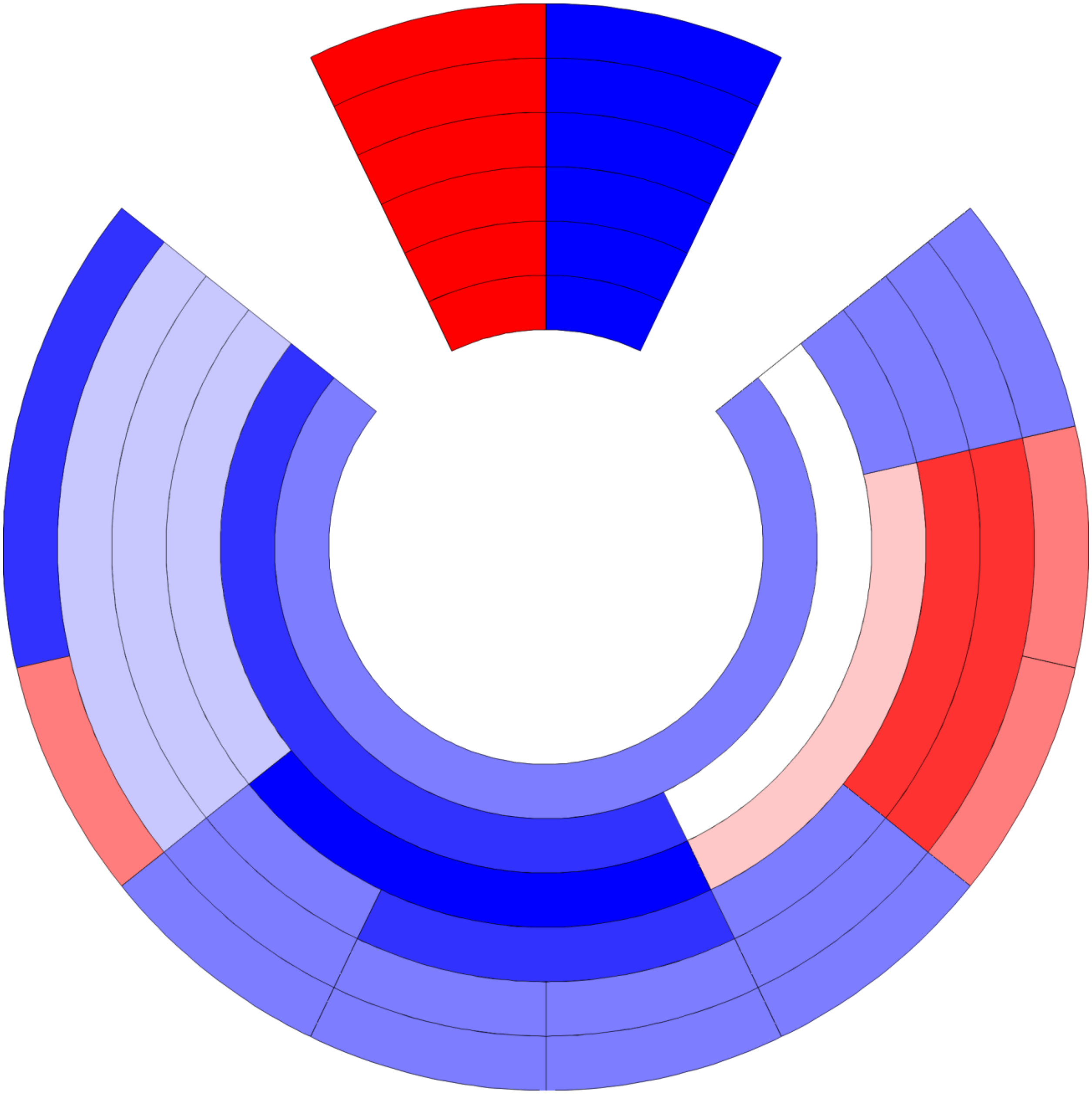

This is the final result of the DHM construction for this small hypothetical dataset that simulated a bottom-up agglomerative clustering approach. In addition to the heat map color limits, the markers at the 0° position show the size of single sequence cluster.

Final Cluster Distribution Table

**Initial**	**0.95**	**0.96**	**0.97**	**0.98**	**0.99**	**1.0**
A	+D	+D	+D	+D	+D	+D
B	A	A	+A	+A	+A	+A
C	I	I	I	I	I	+I
D	F	F	F	+F	+F	+F
E	J	+J	+J	+J	+J	+J
F	G	G	B	G	+G	+G
G	B	B	G	+B	+B	+B
H	E	E	+E	+E	+E	+E
I	C	C	C	C	C	+C
J	H	H	H	H	H	H

"+" indicates the start of a new cluster.

Top-Down Sequence Order Preparation

In the case of the top-down, centroid-based clustering, using arbitrary or aligned sequence ordering is not ideal since they both can result in sequences being binned into clusters that do not contain their closest matching centroid, and to ensure that this is not the case, input order must be staggered from opposite ends of a multiple alignment for each clustering task as shown in Figure 1 of the manuscript, which is also provided below. The multiple alignment can be performed by any multiple alignment program, however the native output will need to be converted to a staggered order with a programming language script.

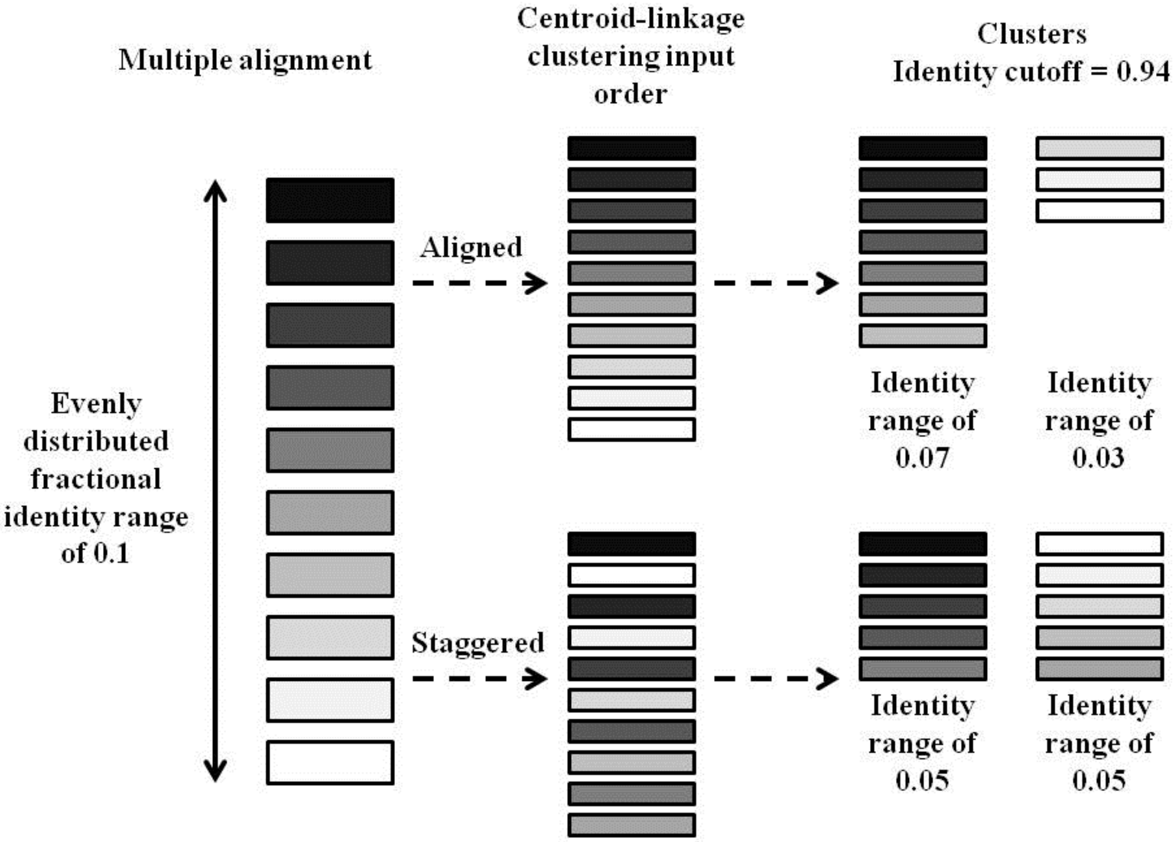

Figure 1. Sequence input ordering. Graphical representation of the binning effect of using alignment-ordered versus staggered sequence input order for "top-down" centroid-based clustering. Shaded rectangles represent sequences, where the shade consistently portrays a specific sequence throughout the diagram. The multiple alignment on the left shows each of the sequences ordered based on fractional identity, where nearby sequences are more closely related than distant ones, and distributed evenly across a fractional identity range of 0.1. For both aligned and staggered input ordering, sequences are read from top to bottom by the UCLUST algorithm of USEARCH and either placed in a cluster that has the best match to the centroid sequence above the given identity cutoff, or is made the centroid sequence of a new cluster if a match cannot be found. In this diagram, centroid sequences are the top sequences of each cluster. With the aligned input order, it is shown that some sequences can be binned in clusters that do not contain their closest centroid match. The staggered input places sequences in correct bins essentially by first defining all centroid sequences.

The DHM construction for the top-down, centroid-based clustering approach is exactly the same as the bottom-up agglomerative approach. However, the sequence staggering step is necessary at each cluster-splitting step from ring to adjacent ring. For this reason, the remainder of this walkthrough will discuss the top-down sequence order preparation steps and skip the DHM construction. We will again discuss the steps for a cutoff range of 0.95-1.0 but for sequences K-T.

To begin, we will perform a multiple alignment of the ten sequences to acquire a tree-output ordering. In our work we used Clustal Omega, however any multiple alignment program that outputs a tree-ordering can be used.

**Unaligned Sequences**	**Aligned Sequences**
Sequence K	Sequence N
Sequence L	Sequence T
Sequence M	Sequence K
Sequence N	Sequence L
Sequence O	Sequence S
Sequence P	Sequence R
Sequence Q	Sequence M
Sequence R	Sequence O
Sequence S	Sequence Q
Sequence T	Sequence P

Once we have obtained our tree-ordered multiple alignment, we can stagger the sequences for optimal centroid-based clustering:

**Aligned Sequences**	**Staggered Sequences**
Sequence N	Sequence N
Sequence T	Sequence P
Sequence K	Sequence T
Sequence L	Sequence Q
Sequence S	Sequence K
Sequence R	Sequence O
Sequence M	Sequence L
Sequence O	Sequence M
Sequence Q	Sequence S
Sequence P	Sequence R

We can now use this staggered order to cluster at a cutoff of 0.95, which in this hypothetical case will yield a single cluster:

0.95

Clustering Cutoff:	0.95
Cluster:	Cluster 1
Sequences:	Sequence N
	Sequence P
	Sequence T
	Sequence Q
	Sequence K
	Sequence O
	Sequence L
	Sequence M
	Sequence S
	Sequence R

We will use this set of sequences from this single 0.95 cutoff cluster as the input for clustering at 0.96. While it is unnecessary to reorder the staggering for the next cutoff in this particular case, we will still show the steps to accurately portray the algorithm that we used. First, we reorder the sequences of the 0.95 cluster according to the original multiple alignment.

Original Aligned Sequences	Reordered Cluster 1
Sequence N	Sequence N
Sequence T	Sequence T
Sequence K	Sequence K
Sequence L	Sequence L
Sequence S	Sequence S
Sequence R	Sequence R
Sequence M	Sequence M
Sequence O	Sequence O
Sequence Q	Sequence Q
Sequence P	Sequence P

Then, we will stagger the reordered sequence just as we did for the 0.95 clustering step. Again, this step may seem unnecessary at this point, but the reasoning behind it will become apparent in later steps.

**Reordered Cluster 1**	**Staggered Cluster 1**
Sequence N	Sequence N
Sequence T	Sequence P
Sequence K	Sequence T
Sequence L	Sequence Q
Sequence S	Sequence K
Sequence R	Sequence O
Sequence M	Sequence L
Sequence O	Sequence M
Sequence Q	Sequence S
Sequence P	Sequence R

We can now use this staggered order to cluster at a cutoff of 0.95:

0.96

Clustering Cutoff:	0.96
Cluster:	Cluster 1
Sequences:	Sequence N
	Sequence T
	Sequence K
	Sequence L
Cluster:	Cluster 2
Sequences:	Sequence P
	Sequence Q
	Sequence O
	Sequence M
	Sequence S
	Sequence R

We will use these sets of sequences from these 0.96 cutoff clusters as the inputs for clustering at 0.97. First, we reorder the sequences of the 0.96 clusters according to the original multiple alignment.

**Original Aligned Sequences**	**Reordered Cluster 1**	**Reordered Cluster 2**
Sequence N	Sequence N	Sequence S
Sequence T	Sequence T	Sequence R
Sequence K	Sequence K	Sequence M
Sequence L	Sequence L	Sequence O
Sequence S		Sequence Q
Sequence R		Sequence P
Sequence M		
Sequence O		
Sequence Q		
Sequence P		

Then, we will stagger the reordered sequence just as we did for the previous clustering step.

**Reordered Cluster 1**	**Staggered Cluster 1**
Sequence N	Sequence N
Sequence T	Sequence L
Sequence K	Sequence T
Sequence L	Sequence K

**Reordered Cluster 2**	**Staggered Cluster 2**
Sequence S	Sequence S
Sequence R	Sequence P
Sequence M	Sequence R
Sequence O	Sequence Q
Sequence Q	Sequence M
Sequence P	Sequence O

We can now use this staggered order to cluster at a cutoff of 0.97:

0.97

Clustering Cutoff:	0.97
Cluster:	Cluster 1
Sequences:	Sequence N
Cluster:	Cluster 2
Sequences:	Sequence L
	Sequence T
	Sequence K
Cluster:	Cluster 3
Sequences:	Sequence S
	Sequence R
	Sequence M
Cluster:	Cluster 4
Sequences:	Sequence P
	Sequence Q
	Sequence O

We will use these sets of sequences from these 0.97 cutoff clusters as the inputs for clustering at 0.98. First, we reorder the sequences of the 0.97 clusters according to the original multiple alignment.

**Original Aligned Sequences**	**Reordered Cluster 1**	**Reordered Cluster 2**
Sequence N	Sequence N	Sequence T
Sequence T		Sequence K
Sequence K		Sequence L
Sequence L		
Sequence S		
Sequence R		
Sequence M		
Sequence O		
Sequence Q		
Sequence P		

**Reordered Cluster 3**	**Reordered Cluster 4**
Sequence S	Sequence O
Sequence R	Sequence Q
Sequence M	Sequence P

Then, we will stagger the reordered sequence just as we did for the previous clustering step.

Reordered	Staggered
**<u>Reordered Cluster 1</u>**	**<u>Staggered Cluster 1</u>**
Sequence N	Sequence N
**<u>Reordered Cluster 2</u>**	**<u>Staggered Cluster 2</u>**
Sequence T	Sequence T
Sequence K	Sequence L
Sequence L	Sequence K
**<u>Reordered Cluster 3</u>**	**<u>Staggered Cluster 3</u>**
Sequence S	Sequence S
Sequence R	Sequence M
Sequence M	Sequence R
**<u>Reordered Cluster 4</u>**	**<u>Staggered Cluster 4</u>**
Sequence O	Sequence O
Sequence Q	Sequence P
Sequence P	Sequence Q

We can now use this staggered order to cluster at a cutoff of 0.98:

0.98

Clustering Cutoff:	0.98
Cluster:	Cluster 1
Sequences:	Sequence N
Cluster:	Cluster 2
Sequences:	Sequence T
	Sequence K
Cluster:	Cluster 3
Sequences:	Sequence L
Cluster:	Cluster 4
Sequences:	Sequence S
	Sequence R
Cluster:	Cluster 5
Sequences:	Sequence M
Cluster:	Cluster 6
Sequences:	Sequence O
	Sequence P
	Sequence Q

We will use these sets of sequences from these 0.98 cutoff clusters as the inputs for clustering at 0.99. First, we reorder the sequences of the 0.98 clusters according to the original multiple alignment.

Original Aligned Sequences	Reordered Cluster 1	Reordered Cluster 2
Sequence N	Sequence N	Sequence T
Sequence T		Sequence K
Sequence K		
Sequence L		
Sequence S		
Sequence R		
Sequence M		
Sequence O		
Sequence Q		
Sequence P		

Reordered Cluster 3	Reordered Cluster 4	Reordered Cluster 5
Sequence L	Sequence S	Sequence M
	Sequence R	

Reordered Cluster 6
Sequence O
Sequence P
Sequence Q

Then, we will stagger the reordered sequence just as we did for the previous clustering step.

Reordered	Staggered
**Reordered Cluster 1**	**Staggered Cluster 1**
Sequence N	Sequence N
**Reordered Cluster 2**	**Staggered Cluster 2**
Sequence T	Sequence T
Sequence K	Sequence K
**Reordered Cluster 3**	**Staggered Cluster 3**
Sequence L	Sequence L
**Reordered Cluster 4**	**Staggered Cluster 4**
Sequence S	Sequence S
Sequence R	Sequence R
**Reordered Cluster 5**	**Staggered Cluster 5**
Sequence M	Sequence M
**Reordered Cluster 6**	**Reordered Cluster 6**
Sequence O	Sequence O
Sequence P	Sequence Q
Sequence Q	Sequence P

We can now use this staggered order to cluster at a cutoff of 0.99:

0.99

Clustering Cutoff:	0.99
Cluster:	Cluster 1
Sequences:	Sequence N
Cluster:	Cluster 2
Sequences:	Sequence T
	Sequence K
Cluster:	Cluster 3
Sequences:	Sequence L
Cluster:	Cluster 4
Sequences:	Sequence S
Cluster:	Cluster 5
Sequences:	Sequence R
Cluster:	Cluster 6
Sequences:	Sequence M
Cluster:	Cluster 7
Sequences:	Sequence O
	Sequence P
	Sequence Q

We will use these sets of sequences from these 0.99 cutoff clusters as the inputs for clustering at 1.0. First, we reorder the sequences of the 0.99 clusters according to the original multiple alignment.

Original Aligned Sequences	Reordered Cluster 1	Reordered Cluster 2
Sequence N	Sequence N	Sequence T
Sequence T		Sequence K
Sequence K		
Sequence L		
Sequence S		
Sequence R		
Sequence M		
Sequence O		
Sequence Q		
Sequence P		

Reordered Cluster 3	Reordered Cluster 4	Reordered Cluster 5
Sequence L	Sequence S	Sequence R

Reordered Cluster 6	Reordered Cluster 7
Sequence M	Sequence O
	Sequence P
	Sequence Q

Then, we will stagger the reordered sequence just as we did for the previous clustering step.

Reordered	Staggered
**<u>Reordered Cluster 1</u>**	**<u>Staggered Cluster 1</u>**
Sequence N	Sequence N
**<u>Reordered Cluster 2</u>**	**<u>Staggered Cluster 2</u>**
Sequence T	Sequence T
Sequence K	Sequence K
**<u>Reordered Cluster 3</u>**	**<u>Staggered Cluster 3</u>**
Sequence L	Sequence L
**<u>Reordered Cluster 4</u>**	**<u>Staggered Cluster 4</u>**
Sequence S	Sequence S
**<u>Reordered Cluster 5</u>**	**<u>Staggered Cluster 5</u>**
Sequence R	Sequence R
**<u>Reordered Cluster 6</u>**	**<u>Staggered Cluster 6</u>**
Sequence M	Sequence M
**<u>Reordered Cluster 7</u>**	**<u>Staggered Cluster 7</u>**
Sequence O	Sequence O
Sequence P	Sequence Q
Sequence Q	Sequence P

We can now use this staggered order to cluster at a cutoff of 1.0:

1.0

Clustering Cutoff:	1.0
Cluster:	Cluster 1
Sequences:	Sequence N
Cluster:	Cluster 2
Sequences:	Sequence T
	Sequence K
Cluster:	Cluster 3
Sequences:	Sequence L
Cluster:	Cluster 4
Sequences:	Sequence S
Cluster:	Cluster 5
Sequences:	Sequence R
Cluster:	Cluster 6
Sequences:	Sequence M
Cluster:	Cluster 7
Sequences:	Sequence O
Cluster:	Cluster 8
	Sequence Q
	Sequence P

These are the final results of the top-down, centroid-based clustering for this small hypothetical dataset.

Final Cluster Distribution Table

**Initial**	**0.95**	**0.96**	**0.97**	**0.98**	**0.99**	**1.0**
K	+N	+N	+N	+N	+N	+N
L	T	T	+T	+T	+T	+T
M	K	K	K	K	K	K
N	L	L	L	+L	+L	+L
O	S	+S	+S	+S	+S	+S
P	R	R	R	R	+R	+R
Q	M	M	M	+M	+M	+M
R	O	O	+O	+O	+O	+O
S	Q	Q	Q	Q	Q	+Q
T	P	P	P	P	P	P

“+” indicates the start of a new cluster.
