# Supplementary figures and images for: Using Dendritic Heat Maps to Simultaneously Display Genotype Divergence with Phenotype Divergence
Source: PLoS One. 2016 Aug 18;11(8):e0161292. doi: 10.1371/journal.pone.0161292 (PMC4990276; doi:10.1371/journal.pone.0161292)

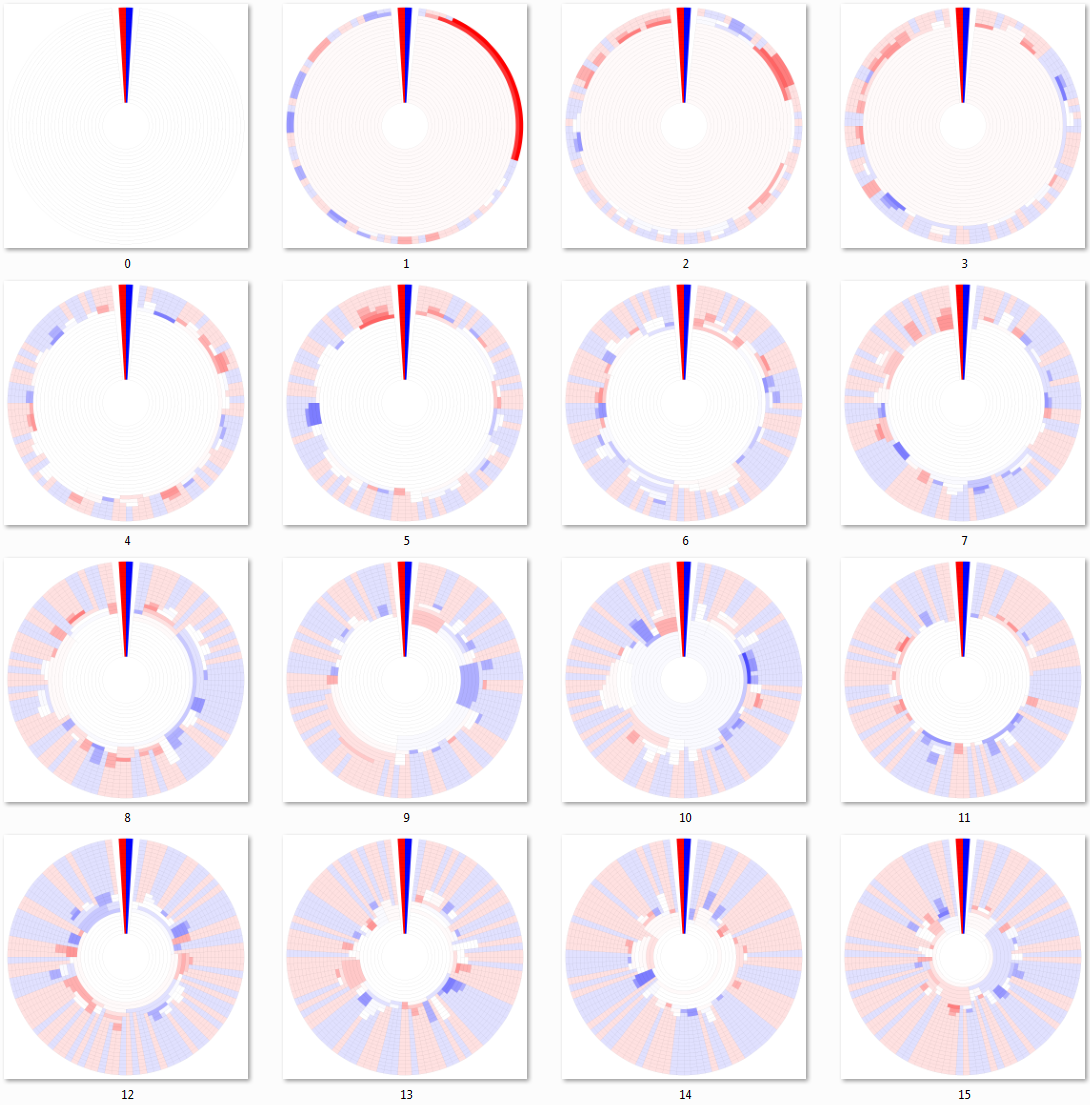

Supplement: S3 File — This file is a zipped folder containing the top-down Perl scripts described in the methods section, high-resolution versions of the images in the discussion, the configuration files used by Circos to plot the images, FASTA files of the artificial datasets, and an Excel file summary of the clusters for each mutation dataset DHM. (ZIP) [file pone.0161292.s003.zip › S3_File/cent.png]
